# Ultrafast Dense Immobilization of Noble Metal Nanoparticles on Customizable Multifunctional Polymer Microspheres for Heterogeneous Catalysis and Multiplexed Biodetection

**DOI:** 10.1002/advs.202520357

**Published:** 2025-12-20

**Authors:** Jie Zhao, Xinyi Liu, Dan Li, Xuefei Jing, Weijie Wu, Liangrui He, Yizhang Tang, Zhen Liu, Yulin Zeng, Haitian Sha, Huibin Qiu, Xujiang Yu, Wanwan Li

**Affiliations:** ^1^ State Key Lab of Metal Matrix Composites, School of Materials Science and Engineering, Zhangjiang Institute for Advanced Study Shanghai Jiao Tong University Shanghai P. R. China; ^2^ School of Materials Science and Engineering Nanjing University of Science and Technology Nanjing Jiangsu P. R. China; ^3^ School of Chemistry and Chemical Engineering, Zhangjiang Institute for Advanced Study, Frontiers Science Center For Transformative Molecules, State Key Laboratory of Metal Matrix Composites Shanghai Jiao Tong University Shanghai P. R. China; ^4^ Inner Mongolia Research Institute of Shanghai Jiao Tong University Huhehot P. R. China

**Keywords:** co‐assembly, heterogeneous catalysts, multiplexed biodetection, noble metal nanoparticles, polymer microspheres

## Abstract

Constructing colloidal particles into designated hierarchies with integrated functionalities offers insights into bottom‐up material fabrication. However, the arbitrary and controllable assembly of individual nanoscale and microscale particles remains highly challenging due to their diverse structural properties. Here, a general paradigm for the robust co‐assembly of plasmonic noble metal nanoparticles and multifunctional polymer microspheres through thermodynamically driven heterocoagulation and coordination interactions is developed. Monodisperse poly(styrene‐co‐maleic anhydride) microspheres (PSMA MSs) are facilely synthesized using a one‐step emulsification method with optional simultaneous encapsulation of magnetic and fluorescent nanoparticles. Upon surfactant removal, the purified PSMA MSs become metastable and trigger ultrafast co‐assembly of silver nanocubes (AgNCs) onto MSs within 5 min with the maximum coverage rate reaching nearly 50%. The densely immobilized AgNCs outside magnetic MSs exhibit excellent catalytic efficiency (>89% conversion across 15 cycles) and enable enzyme integration for cascade reactions. Moreover, the largest reported plasmonic‐fluorescent encoded array (124 codes) is realized, achieving a significantly improved detection limit of 10 copies/reaction for respiratory viruses. This work addresses the long‐standing challenge of achieving uniform distribution and dense immobilization of plasmonic noble metal nanoparticles and offers a general approach to fabricating designated hierarchies based on noble metal nanoparticles, with integrated functionalities for high‐performance practical applications.

## Graphical abstract

This work proposes a facile co‐assembly strategy to achieve highly uniform and dense immobilization of diverse noble metal nanoparticles onto metastable multifunctional (e.g., magnetic or fluorescent) polymer microspheres, enabling fabrication of complex hierarchical superstructures. The representative silver nanocube‐decorated poly(styrene‐co‐maleic anhydride) microspheres achieve excellent heterogeneous catalytic performance, as well as highly sensitive and high‐throughput virus biodetection.

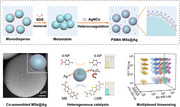

## 1 Introduction

The assembly of diverse functional particles into multifunctional superstructures has attracted widespread interest due to their distinctive properties and collective functionalities (e.g., structural, magnetic, and optical), which surpass those of individual components [1, 2, 3, 4]. Such superstructures have exhibited tremendous potential in catalysis, energy storage, optoelectronic devices, antibacterial, biosensing, diagnostic platforms, and drug delivery [5, 6, 7, 8, 9]. Among these, noble metal nanoparticles (NMNPs)‐based hierarchical superstructures are particularly promising due to their unique localized surface plasmon resonance (LSPR) and capability to integrate with other functionalities [10, 11, 12, 13]. However, the aggregation tendency of NMNPs [14, 15], combined with the scarcity and high‐cost of noble metals, poses significant challenges to constructing advanced multifunctional superstructures [16, 17, 18].

Polymer microspheres (MSs), characterized by large outer surface areas and tunable internal structures, as well as high bioactivity, serve as ideal matrices for NMNPs immobilizing (e.g., Au, Ag, Pt) and/or co‐assembling diverse functional nanoparticles [19, 20, 21, 22, 23, 24, 25]. Over the past decades, various strategies have been established to enhance the immobilization efficiency and uniformity of NMNPs distribution, such as in‐situ reduction, electrostatic adsorption, covalent coupling, layer‐by‐layer assembly, and interfacial self‐assembly, tailored to the practical surface properties of different types of microspheres [19, 20, 21, 26, 27]. Despite some successes, these strategies still suffer from low NMNPs loading efficiency, uneven nanoparticle distribution, and morphological nonuniformity [22, 23]. Meanwhile, functional particles (e.g., Fe_3_O_4_ or quantum dots) can be typically encapsulated via microsphere swelling or suspension polymerization [28, 29, 30]. Furthermore, multi‐step preparation processes frequently lead to polydisperse assemblies, imprecise component arrangement, and inefficient functional integration [24]. Recent advances in membrane emulsification/solvent evaporation techniques have enabled the one‐step synthesis of highly uniform functional microspheres without requiring chemical modifications [31]. This process involves the formation of homogeneous emulsion droplets containing functional nanoparticles and polymer chains in the presence of surfactants, followed by solvent evaporation‐induced solidification to produce engineered microspheres with superior homogeneity and controllability [32]. As one of the most commercially viable methods, membrane emulsification also stands out for its ability to produce monodisperse microspheres with embedded magnetic or fluorescent nanoparticles by simply controlling pore size and external pressure [33]. However, the heavy reliance on surfactants in membrane emulsification has inversely rendered microspheres that are inaccessible for subsequent surface functionalization with other components [34, 35], particularly the highly anticipated surface immobilization of NMNPs. Therefore, investigating the surface properties of polymer microspheres will be crucial for the precise synthesis of customizable, NMNPs‐decorated multifunctional microspheres and hierarchical superstructures.

Herein, we report a series of unprecedented densely immobilized NMNPs‐based hierarchical superstructures achieved through precise engineering of the structure and surface properties of polymer microspheres. Various polymer microspheres, including poly(styrene‐co‐maleic anhydride) (PSMA), polylactic acid, polycaprolactone, and polystyrene MSs, were synthesized using the Shirasu porous glass (SPG) membrane emulsification method with the sodium dodecyl sulfate (SDS) surfactants as the stabilizer. Complete surfactant removal enabled pure PSMA microspheres to undergo rapid co‐assembly with silver nanocubes (AgNCs; average sizes: 25.4, 32.6, 48.3, and 72.0 nm), achieving uniform dispersion and dense immobilization. Mechanistic studies revealed that this spontaneous co‐assembly was driven by thermodynamic heterocoagulation, further stabilized by coordination interactions between PSMA and silver atoms, a mechanism applicable to various polymer MSs and NMNPs. The resulting dense and evenly immobilized AgNC shells exhibited excellent LSPR properties, abundant active sites, and strong metal‐enhanced fluorescence (MEF). Notably, the SPG method also facilitated spatially confined encapsulation of multiple functional units within a single hybrid system, such as magnetic nanoparticles and fluorescent quantum dots. These plasmonic‐magnetic superstructures demonstrated excellent catalytic efficiency for organic transformations, with over 89% conversion efficiency across 15 catalytic cycles, as well as successful GOx enzyme conjugation for cascade reactions. Furthermore, we developed the largest reported plasmonic‐fluorescent encoded array (124 codes) with a 20‐fold fluorescence enhancement, enabling highly sensitive multiplexed detection of respiratory viruses with a significantly improved detection limit of 10 copies per reaction. These findings establish a surfactant‐mediated strategy for on‐demand fabrication of uniform, multifunctional microspheres with precisely controlled component integration for diverse practical applications.

## 2 Results and Discussion

### 2.1 Ultrafast Dense Immobilization of NMNPs Onto PSMA MSs

**FIGURE 1:**
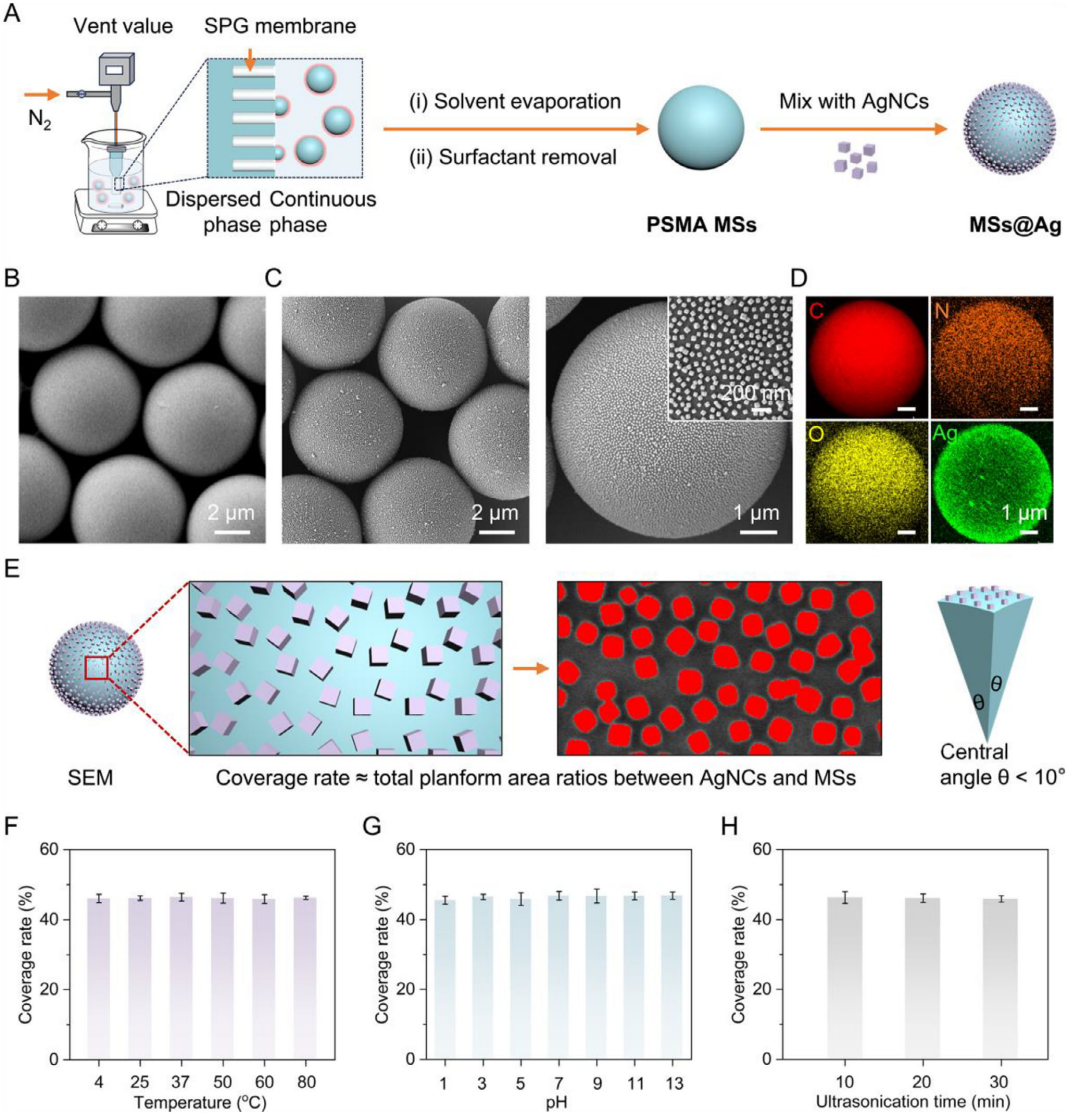
Synthesis and characterization of AgNCs‐densely‐immobilized PSMA MSs. (A) Schematic illustration of the synthesis process. (B) SEM image of PSMA MSs prepared via SPG membrane emulsification. (C) SEM image of the co‐assembly between 6.4 µm PSMA MSs and 48.3 nm AgNCs at a 31.3:1 mass ratio. (D) EDS mappings of representative MSs@Ag between 6.4 µm PSMA MSs and 48.3 nm AgNCs at a 31.3:1 mass ratio. (E) Schematic illustration of the calculation of the defined coverage rate on the PSMA MSs surface. (F‐H) Coverage rates under different conditions: temperatures (F), pH conditions (G), and ultrasound durations (H).

NMNPs‐densely‐immobilized PSMA MSs were achieved by synthesizing monodisperse MSs followed by mixing with NMNPs (Figure 1A). PSMA, a petroleum‐based biocompatible copolymer, exhibits a spaghetti‐shaped length‐tunable linear structure, excellent physicochemical stability under various reaction conditions, and inert surface chemical properties (Figure S1). Moreover, its inherent maleic anhydride moiety allows facile functionalization for broad biomedical applications. Therefore, PSMA was selected as the representative matrix for fabricating polymer MSs. During the synthesis of PSMA MSs, SDS served as a stabilizer. Following the typical SPG membrane emulsification process, the dispersed phase containing 62.5 mg mL^−1^ PSMA free chains (Mw: 224 000, 7 wt.% anhydride) in toluene was extruded through a hydrophobic SPG membrane into the continuous phase (5 mg mL^−1^ SDS aqueous solution) under nitrogen pressure. Subsequently, the emulsion was stirred to evaporate toluene, causing the droplets to solidify into SDS‐stabilized PSMA MSs. The as‐synthesized products were then collected and washed to obtain PSMA MSs (referred to as SDS‐free MSs unless otherwise specified).

We first synthesized monodisperse PSMA MSs with an average diameter of 6.4 ± 0.4 µm to investigate the significant role of the surfactant in manipulating the surface properties of PSMA MSs and promoting the co‐assembly process (Figure 1B; Figure S2). Ag nanocubes (AgNCs) with superior LSPR properties were adopted as model NMNPs in this work. The AgNCs with an average size of 48.3 ± 4.4 nm were prepared by a modified polyol reduction method (Figure S3) [36]. As shown in Figure 1C, mixing 6.4 µm PSMA MSs and 48.3 nm AgNCs at a mass ratio of 31.3:1 in H_2_O triggered co‐assembly within a short period of 5 min. Scanning electron microscopy (SEM) images revealed that AgNCs were uniformly distributed on the PSMA MSs (denoted as MSs@Ag hereafter). Energy‐dispersive spectroscopy (EDS) mapping results confirmed the complete removal of SDS from the PSMA MSs (Figure S4 and Table S1) and demonstrated a uniform distribution of C, N, O, and Ag (Figure 1D), indicating successful co‐assembly of AgNCs onto the PSMA MSs with dense immobilization. In contrast, SDS‐stabilized PSMA MSs exhibited negligible AgNCs adsorption (Figures S5 and S6, and Table S2). These results indicate that SDS not only plays a critical role in stabilizing emulsion droplets but also modulates the surface properties of microspheres.

To quantify the relative abundance and immobilization density of co‐assembled AgNCs on the outer surface of the MSs under different conditions, we defined a semi‐quantitative metric termed “coverage rate” (Figure 1E). This parameter was calculated as the ratio of the total projected area of AgNCs to that of MSs within a defined cross‐section of the MSs@Ag. To ensure mathematical accuracy, the randomly selected cross‐sections were constrained to a central angle of less than 10°, and the coverage rates were determined from at least three independent cross‐sections per condition. Based on this analysis, the amount of 48.3 nm AgNCs co‐assembled onto 6.4 µm PSMA MSs reached equilibrium within approximately 5 min, achieving a coverage rate of 46.5%, with no significant changes observed even after extending the mixing time to 30 min (Figure S7). Furthermore, MSs@Ag exhibited excellent stability, maintaining constant coverage rates under different conditions, such as a wide temperature range from 4 to 80 °C (Figure 1F; Figure S8), a broad range of pH values from 1 to 13 (Figure 1G; Figure S9), and six widely used buffer solutions with different ionic species and strengths (Figure S10). Notably, coverage rates remained unchanged even after the MSs@Ag were being treated with ultrasonication for 30 min (Figure 1H; Figure S11) or stored in an aqueous solution for 90 days (Figure S12).

### 2.2 Robust Dense Immobilization Behavior Across Varied Parameters

**FIGURE 2:**
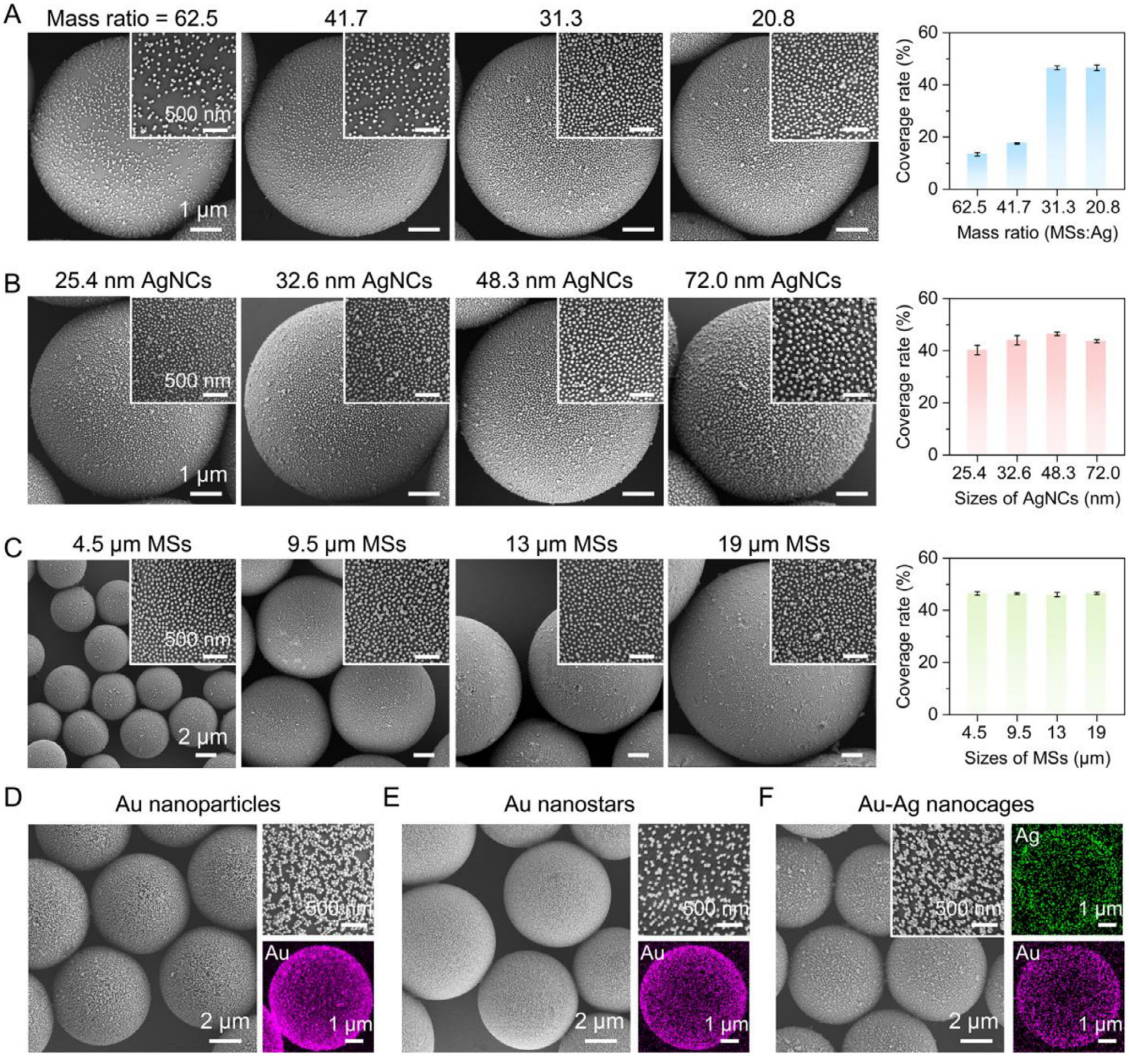
Robust dense co‐assembly and the versatility of MSs@NMNPs superstructures. (A) SEM images and coverage rates of 48.3 nm AgNCs on 6.4 µm PSMA MSs at different PSMA MSs versus AgNCs mass ratios of 62.5, 41.7, 31.3, and 20.8. Inserts are high‐resolution SEM images. (B) SEM images and coverage rates of differently sized AgNCs (25.4, 32.6, 48.3, and 72.0 nm AgNCs) on 6.4 µm PSMA MSs. (C) SEM images and coverage rates of 48.3 nm AgNCs on differently sized PSMA MSs (4.5, 9.5, 13, and 19 µm). Inserts are high‐resolution SEM images. (D–F) SEM images and element mappings of PSMA MSs@Au nanoparticles (D), PSMA MSs@Au nanostars (E), and PSMA MSs@Au‐Ag nanocages (F).

To evaluate the feasibility and versatility of this facile and robust co‐assembly strategy, we first investigated the effect of different mixing mass ratios of 6.4 µm PSMA MSs and 48.3 nm AgNCs. As shown in Figure 2A and Figure S13, robust co‐assembly with uniformly distributed AgNCs was observed across a wide range of mass ratios from 125:1, 62.5:1, 41.7:1, 31.3:1 to 20.8:1. The surface density of AgNCs on PSMA MSs exhibited a positive correlation with the mass ratio, yielding coverage rates ranging from 9.5% to 46.5%. Notably, no further increase in the coverage rate was observed by increasing the amount of AgNCs from 31:1 to 20.8:1, indicating the maximum coverage approached 46.5% or less than 50%, even in the presence of excessive AgNCs. We believe this to be mainly attributed to the steric hindrance of PVP outside of AgNCs and the electrostatic repulsion between adjacent AgNCs as they also maintained good dispersion in solution [37]. Next, we examined the influence of particle size on co‐assembly behavior. PSMA MSs with average diameters of 4.5 ± 0.4, 9.5 ± 0.6, 13 ± 0.9, and 19 ± 1.3 µm were prepared by employing SPG membranes with different pore sizes and adjusting the emulsion extruding speeds. Similarly, AgNCs with average sizes of 25.4 ± 3.4, 32.6 ± 3.2, and 72.0 ± 4.5 nm were synthesized via the polyol reduction method (Figure S14). Remarkably, when mixed with 6.4 µm PSMA MSs, AgNCs of all sizes exhibited spontaneous and robust co‐assembly, forming dense, uniform shells regardless of their dimensions (Figure 2B). The resulting coverage rates reached up to 40.2%, 44.0%, 46.5%, and 43.6%, respectively. Analogously, varying the size of PSMA MSs, dense and uniform distribution of the 48.3 nm AgNCs was achieved, maintaining consistent coverage rates of approximately 46% (Figure 2C). To further demonstrate the generality of this approach, we extended the co‐assembly to Au nanoparticles, Au nanostars, and bimetallic Au‐Ag nanocages (Figures S15–S17), with average sizes of 50, 45, and 48 nm, respectively. All three kinds of NMNPs spontaneously assembled onto the 6.4 µm PSMA MSs (Figure 2D–F), forming similarly dense monolayers. EDS mapping results clearly confirmed the distinct distributions of Au and/or Ag in the resulting composites with analogous NMNP shells. This observation further verified the generality and high efficiency of this co‐assembly strategy, which has never been achieved by other methods.

### 2.3 Mechanism of the Co‐Assembled MSs@Ag Composites

**FIGURE 3:**
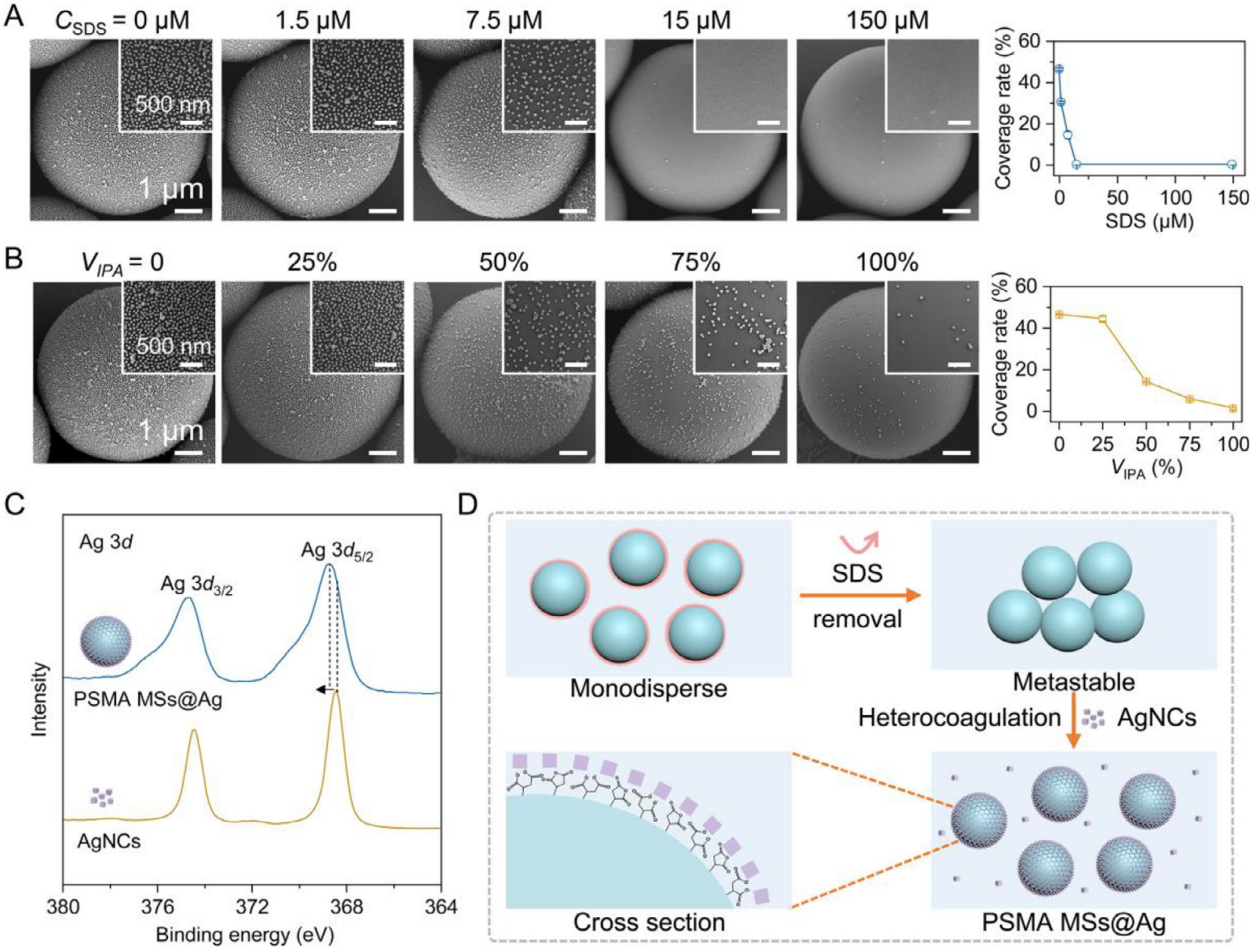
Mechanism of the co‐assembled MSs@Ag. (A) SEM images and coverage rates of the MSs@Ag co‐assembled by tuning the concentrations of SDS (0, 1.5, 7.5, 15, and 150 µM). Inserts are high‐resolution SEM images. (B) Effect of solvent polarity (the volume percentage of 2‐propanol in water, *V*_IPA_ = 0, 25%, 50%, 75%, and 100%). Inserts are high‐resolution SEM images. (C) The high‐resolution XPS spectra of the Ag 3*d* orbital of AgNCs and PSMA MSs@Ag. (D) Representation of heterocoagulation and coordination interactions between AgNCs and PSMA MSs.

Impressed by this spontaneous and stable formation behavior, we then investigated the underlying co‐assembly mechanisms. To elucidate the surfactant's role, we first introduced different amounts of additional SDS into the aqueous suspension of PSMA MS prior to adding AgNCs. Notably, even a low concentration of supplemental SDS (about 15 µM) significantly inhibited the co‐assembly process between 6.4 µm PSMA MSs and 48.3 nm AgNCs (Figure 3A; Figure S18). X‐ray photoelectron spectroscopy (XPS) analysis revealed the adsorption of SDS onto the outer surface of PSMA MSs with the gradual increase of characteristic Na and S signals (Figure S19), demonstrating the leading role of high surface intension of PSMA MSs after surfactant removal and the crucial role of AgNCs to decrease the surface intension. Quantitative analysis demonstrated an inverse correlation between coverage rate and SDS concentration, with a sharp decrease observed at about 15 µM. Interestingly, pre‐formed MSs@Ag maintained structural stability in SDS‐containing aqueous solutions (Figure S20). These findings conclusively indicate that the absence of the SDS surfactant is crucial for initiating the co‐assembly process.

To investigate surfactant removal effects, we conducted Zeta potentials and dynamic light scattering (DLS) measurements on 6.4 µm PSMA MSs before and after SDS removal. The SDS‐stabilized PSMA MSs exhibited a strong negative Zeta potential of ‐108.9 mV with a mean hydrated diameter of 7.4 µm (Figures S21 and S22). In stark contrast, after complete SDS removal, the Zeta potential shifted to −28.5 mV while the mean hydrated diameter increased to 8.1 µm, accompanied by an elevated polydispersity index of 0.34. Notably, the SDS‐free PSMA MSs displayed pronounced aggregation tendencies and strong adhesion to glass surfaces when resuspended in water (Figure S23). This phenomenon demonstrated the much stronger surface tension between the SDS‐free PSMA MSs than the electrostatic repulsion force despite a relatively large Zeta potential of −28.5 mV, which in turn made the MSs unstable. Moreover, given that the Zeta potential of AgNCs is nearly zero, the dominating surface tension drives the assembly process regardless of the repulsive force. These observations indicate that SDS removal induces metastability in the polymer microspheres by reducing interfacial tension, which thermodynamically drives rapid, spontaneous co‐assembly of AgNCs onto PSMA MSs. This mechanism is consistent with the classical heterocoagulation phenomenon between particles with significant size disparities [38, 39]. The thermodynamically driven heterocoagulation was further supported by a solvent polarity‐correlated co‐assembly behavior (Figure 3B; Figure S24). Reduced solvent polarity diminished the interfacial tension of metastable PSMA MSs, effectively slowing or preventing the heterocoagulation process. Conversely, temperature variations (0‐80 °C) showed minimal influence on interfacial tension but only affected particle mobility, maintaining consistent AgNC coverage rates of ∼44.9% with only minor fluctuations (Figure S25). Notably, the 48.3 nm AgNCs exhibited a negatively charged Zeta potential of −6.9 mV, consistent with the charge‐independent nature of heterocoagulation. This phenomenon was further corroborated using other negatively charged MSs (Figure S26), including polylactic acid MSs, polycaprolactone MSs, and polystyrene MSs. High‐resolution XPS analysis of the Ag 3*d* orbital revealed distinct electronic states between pristine AgNCs and PSMA MSs@Ag (Figure 3C; Figure S27). The pristine AgNCs exhibited a characteristic metallic silver peak with the Ag 3*d*_5/2_ binding energy (BE) of 368.4 eV [40, 41], whereas the PSMA MSs@Ag sample displayed a significant shift of Ag 3*d*_5/2_ by + 0.3 eV to about 368.7  eV. This binding energy shift of Ag 3*d* BE in PSMA MSs@Ag indicates the presence of strong coordination interactions between PSMA and Ag atoms (Ag‐O bonding) and the positive role of the well‐uniform monodispersed state of AgNCs on the MS surface [42, 43]. Besides, the observed red shift of the characteristic C = O stretching vibration peaks from 1858 and 1780 cm^−1^ to 1850 and 1774 cm^−1^ in PSMA after co‐assembling with AgNCs further confirmed the existence of coordination interactions (Figure S28) [44, 45, 46]. Collectively, these findings demonstrate that SDS removal triggers the spontaneous, rapid, and stable co‐assembly between metastable MSs and AgNCs through synergistic heterocoagulation and underlying coordination interactions (Figure 3D).

### 2.4 Plasmonic‐Magnetic MSs@Ag Heterogeneous Catalysts

**FIGURE 4:**
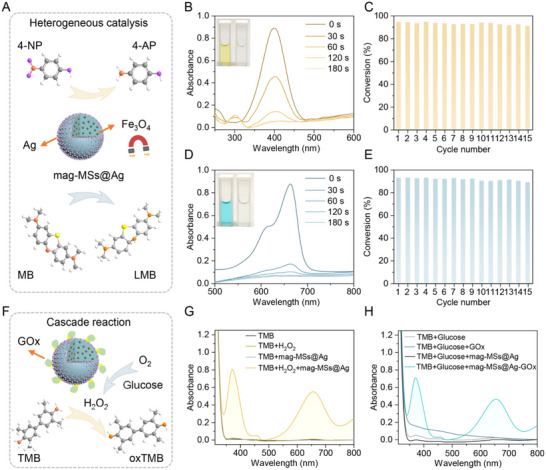
Plasmonic‐magnetic MSs@Ag as heterogeneous catalysts. (A) Schematic illustration of the reduction reaction of 4‐NP and MB catalyzed by mag‐MSs@Ag. (B) Time‐dependent UV–vis spectra of 4‐NP solution using mag‐MSs@Ag as a catalyst. The solution color changed from yellow to colorless (inset photographs). (C) Recyclability test for mag‐MSs@Ag for the reduction of 4‐NP. (D) Time‐dependent UV‐vis spectra of MB solution using mag‐MSs@Ag as a catalyst. The solution color changed from blue to colorless (inset photographs). (E) Recyclability test for mag‐MSs@Ag for the reduction of MB. (F) Illustration of the GOx enzyme‐mediated cascade reaction. (G) UV–vis spectrum of the oxidation of TMB catalyzed by mag‐MSs@Ag. (H) UV–vis spectrum for the cascade reaction sequentially catalyzed by mag‐MSs@Ag‐GOx.

Having demonstrated the uniform and stable immobilization of AgNCs, we next evaluated their catalytic performance in heterogeneous reactions. To enable sustainable and magnetically controllable catalysis, we incorporated 10 nm Fe_3_O_4_ nanoparticles into PSMA MS (denoted as mag‐MSs@Ag) via SPG membrane emulsification, followed by a similar co‐assembly procedure of AgNCs (Figure 4A). The mag‐MSs@Ag exhibited excellent heterogeneous catalytic performance, achieving over 95.5% conversion of 4‐nitrophenol (4‐NP) to 4‐aminophenol (4‐AP) (Figure 4B) and 93.3% conversion of methylene blue (MB) to leucomethylene blue (LMB) (Figure 4D) within a residence time of 3 min. Control experiments confirmed the essential catalytic role of mag‐MSs@Ag, as reactions with only use of sodium borohydride (NaBH_4_) showed negligible conversion of 4‐NP (Figure S29) and MB (Figure S30). Moreover, the magnetic responsiveness enabled facile catalyst recovery and reuse, with over 89% efficiency maintained over 15 cycles (Figure 4C,E). The robust, durable, and recyclable catalytic properties outperformed other currently reported NMNP‐decorated catalysts (Table S3) [22, 34, 47, 48, 49, 50, 51]. The superior catalytic performance was mainly attributed to the dense and uniform AgNC shell formed by the rapid and efficient co‐assembly, demonstrating the unique advantages of our approach in fabricating designated hierarchies with integrated functionalities through one‐step SPG membrane emulsification and facile co‐assembly. Multistep cascade reactions were further explored, building on the enzyme‐binding capability of NMNPs. As a proof‐of‐concept demonstration, we immobilized glucose oxidase (GOx) onto AgNCs through thioctic acid‐EDC/NHS chemistry, yielding mag‐MSs@Ag‐GOx composites. This configuration enabled a two‐step cascade process, the oxidation of glucose catalyzed by GOx and the subsequent reaction of the generated H_2_O_2_ with 3,3’,5,5’‐tetramethylbenzidine (TMB) by AgNCs (Figure 4F). As shown in Figure 4G,H, mag‐MSs@Ag easily oxidized TMB upon addition of exogenous H_2_O_2_, showing two characteristic absorption peaks at 370 and 652 nm, but failed to oxidize TMB in the presence of glucose. In stark contrast, strong characteristic peaks were observed by adding mag‐MSs@Ag‐GOx into the mixture of glucose and TMB, demonstrating the functional efficacy of this engineered co‐assembled structure for multistep enzymatic‐metal catalytic processes.

### 2.5 Plasmonic MSs@Ag With Enhanced Fluorescence Effect

**FIGURE 5:**
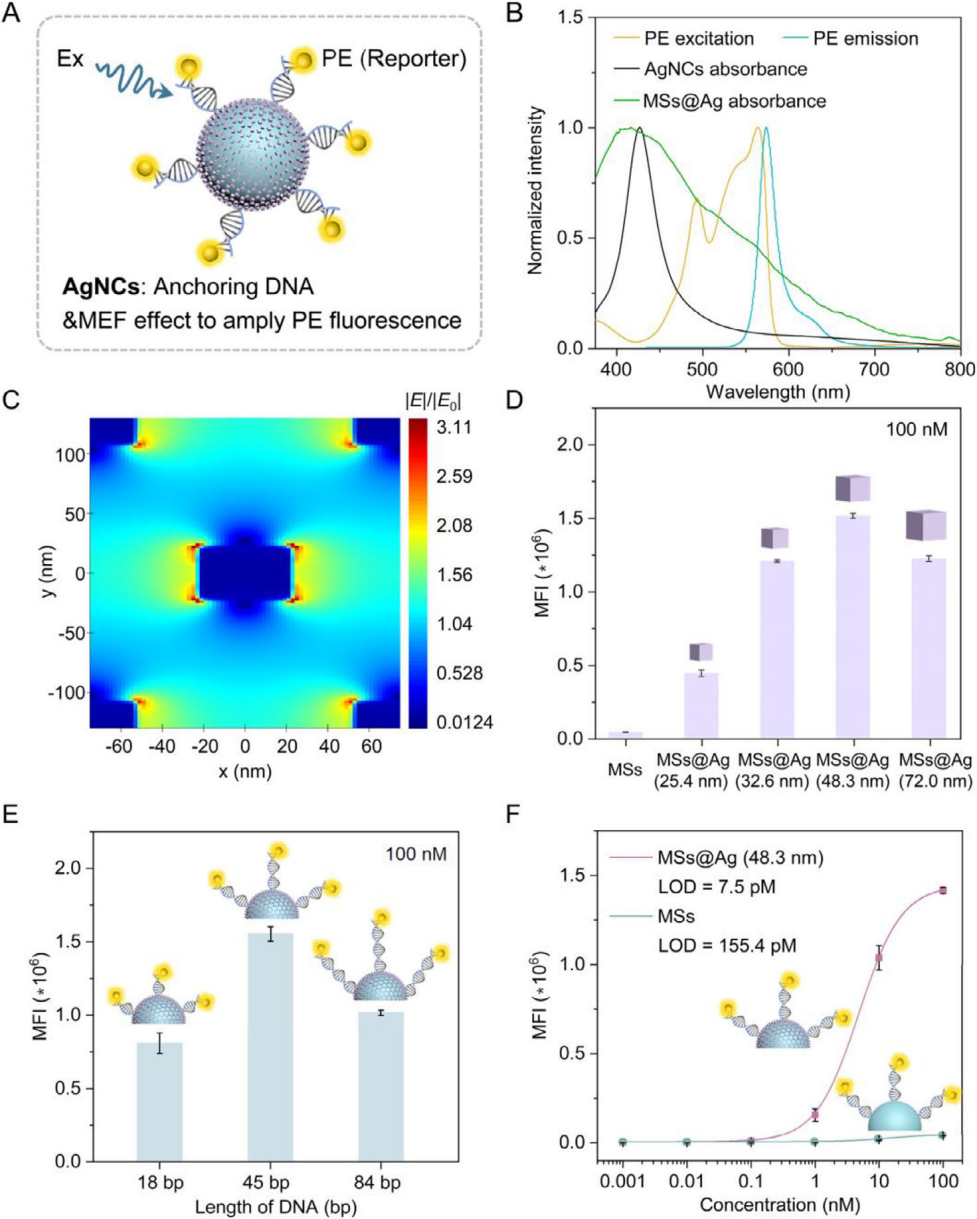
Plasmonic MSs@Ag with enhanced fluorescence effect. (A) Schematic of the MSs@Ag anchoring DNA and MEF effect to amply fluorescence. (B) Normalized absorption spectra of the PSMA MSs@Ag, excitation and emission spectra of the PE. (C) Local electric field of AgNCs on the PSMA MSs. (D) MFI values detected at different AgNCs decorated MSs (100 nM of 45 bp targeted ssDNA). (E) Different DNA lengths on MFI of MSs@Ag‐48.3 nm. (18, 45, and 84 bp; 100 nM of each targeted ssDNA). (F) Comparison of the sensitivities using MSs@Ag‐48.3 nm and MSs (45 bp targeted ssDNA).

NMNPs are of particular value for their ability to enhance fluorophore intensity through MEF via strong electromagnetic fields [52, 53]. Among these, AgNCs exhibit exceptional MEF effects due to their intense visible‐range surface plasmon resonance. Besides, AgNCs can also conjugate with the complementary single‐stranded DNA (capture ssDNA) to target specific DNA fragments (targeted ssDNA) in samples via Ag‐thiolate interactions. After recognizing the targeted DNA fragments, streptavidin‐R‐phycoerythrin (SAPE) fluorescent reporters label the resulting double‐stranded DNA through biotin‐streptavidin binding [54]. We first investigated the MEF of PE mediated by AgNCs immobilized on the surfaces of MSs (Figure 5A). As shown in Figure 5B, the free 48.3 nm AgNCs exhibited only a narrow absorption peak centered at 427 nm, showing minimal spectral overlap with PE's excitation or emission profiles. In contrast, the assembled AgNC shells on MSs showed a broadening of the surface plasmon resonance ranging from 375 to 700 nm [55], fully covering PE's excitation and emission spectra. This broadband coverage demonstrates the unique optical properties arising from the uniform distribution of AgNCs and suggests strong MEF potential for detection enhancement. The finite‐difference time‐domain (FDTD) simulation shows that immobilizing AgNCs on PSMA MS induces a stronger electric field at their corners or tips (Figure 5C and Movie S1), highlighting the positive effect of the dense AgNC shell. This phenomenon was in good consistency with the behavior of anisotropic nanoparticles reported in the literature [56, 57]. Notably, the 48.3 nm AgNCs‐decorated MSs showed the highest median fluorescence intensity (MFI) compared to those with 25.4, 32.6, and 72.0 nm AgNCs (Figure 5D), indicating a size‐dependent relationship between the MEF and AgNCs. We attributed this to the balance between AgNCs’ exposed surface area for anchoring capture ssDNA and their coverage density on MSs. Since MEF intensity depends critically on the distance between NMNPs and fluorophores [52, 58], we quantitatively measured the separation distance between AgNCs and PE. Figure 5E reveals that the MFI strongly correlates with the length of the DNA sequence (details shown in Table S4). The optimized length is ∼45 base‐pairs (about 16.5 nm), consistent with the theoretical predictions of < 30 nm [23]. The optimized MSs@Ag‐48.3 nm showed exceptional detection sensitivity, with the limit of detection (LOD) value of about 7.5 pM compared to the values achieved by MSs@Ag‐25.4 nm (22.2 pM), MSs@Ag‐32.6 nm (15.5 pM), and MSs@Ag‐72.0 nm (12.3 pM) (Figure S31). Remarkably, this is a 20‐fold improvement over undecorated MSs (155.4 pM), as confirmed by calibration curves (Figure 5F). These results conclusively demonstrate the superior MEF performance of AgNCs‐decorated MSs for highly sensitive multiplexed biodetection.

### 2.6 Plasmonic‐Fluorescent Encoded QDs‐MSs@Ag Array for Multiplexed Respiratory Viruses Detection

**FIGURE 6:**
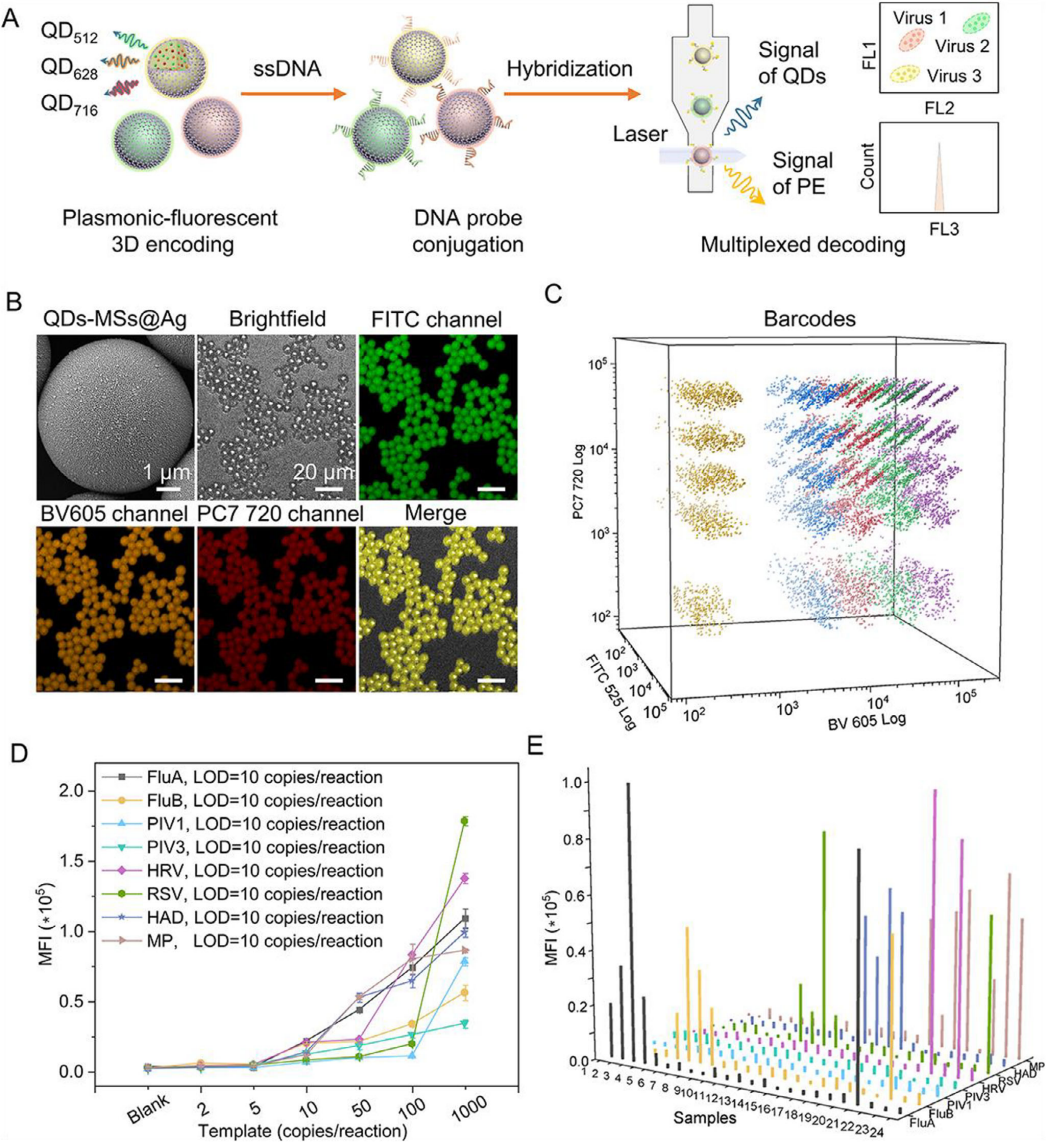
Plasmonic‐fluorescent encoded QDs‐MSs@Ag array. (A) Schematic of QDs‐MSs@Ag barcodes for the multiplexed diagnosis of respiratory viruses. (B) SEM and laser scanning confocal microscopy images of QDs‐MSs@Ag encoded with QD_512_, QD_628_, and QD_716_. (C) 3D diagram of Ag‐decorated QDs‐based barcode matrix array (124 codes) encoded by color (QD_512_, QD_628_, and QD_716_) and intensity (5 levels). (D) Sensitivity of QDs‐MSs@Ag barcodes for capturing corresponding respiratory viruses. (E) Verification of QDs‐MSs@Ag barcodes toward 24 clinical samples.

Multiplexed detection, which refers to the simultaneous analysis of multiple biomarkers, is essential for biotechnology and clinical diagnostics [59, 60, 61, 62]. Various fluorescent biosensing techniques enable highly sensitive and high‐throughput biodetection [63]. To further improve throughputs, fluorescent microspheres incorporating quantum dots (QDs), often termed as QD‐encoded MSs, have been developed. We propose that the co‐assembled AgNCs‐decorated QD‐encoded MSs can simultaneously meet both key requirements for multiplexed detection. To this end, we designed a plasmonic‐fluorescent encoded QDs‐MSs@Ag array, immobilizing AgNCs on the outer MS surface while encapsulating diverse QDs internally (Figure 6A). AgNCs not only enhance fluorescence via MEF but also capture DNA probe, while internally encapsulated QDs significantly expand the encoding capacity through distinct spectral signatures. We then evaluated the capacity of the internal space of MSs to encapsulate fluorescent materials, enhancing detection throughput alongside the previously demonstrated surface‐assembled AgNCs for detection sensitivity enhancement. We chose environmentally benign indium phosphide (InP) QDs, a class of fluorescent probes widely used in various important fields [64]. Motivated by this, we adopted three types of InP QDs with distinct emission peaks at 512 nm (denoted as QD_512_), 628 nm (denoted as QD_628_), and 716 nm (denoted as QD_716_) (Figures S32–S34) as the representative QDs for the following studies. Following a one‐step SPG membrane emulsification process, QDs were precisely encapsulated into MSs at controlled concentrations in toluene/PSMA mixtures, generating QD‐encoded MSs. As shown in Figure 6B, the SEM image confirmed that a simple mixture of QDs‐encoded MSs with AgNCs yielded AgNCs‐densely‐immobilized QDs‐encoded MSs (denoted as QDs‐MSs@Ag). A series of luminescence images captured via laser scanning confocal microscopy (LSCM) depict the representative QDs‐MSs@Ag incorporated with a mixture of QD_512_, QD_628_ and QD_716_. The LSCM images demonstrated well‐overlapped fluorescence signals of all three QDs. These results confirmed uniform encapsulation of fluorescent nanoparticles within MSs based on SPG membrane emulsification. Notably, the decoration of AgNCs significantly enhanced the optical stability of QDs under harsh conditions (Figure S35), such as acidic environments (e.g., pH = 1) or elevated temperatures (e.g., 60 °C), whereas the fluorescence of QDs encapsulated inside QDs without AgNC shell protection decreased apparently under these conditions (Figure S36).

Building on these advantages, we constructed a large 3D QDs‐based barcode matrix array encoded by both color (QD_512_, QD_628_, and QD_716_) and intensity (5 levels) (Figure S37). This represents the largest 3D NMMP‐decorated QDs‐based barcode matrix array reported to date, with a coding capacity of 5 × 5 × 5‐1 (Figure 6C; Figure S38 and Table S5) [18, 65, 66]. Although the AgNC shell caused a measurable fluorescence intensity reduction in the 3D QDs‐MSs@Ag array compared to that of the 3D QDs‐MSs array due to the photon blocking by AgNCs (Figure S39), all adjacent clusters maintained highly distinguishable spatial coordinates. These results demonstrate the significant potential of our combined SPG membrane emulsification and co‐assembly strategy for practical encoding applications.

To validate the multiplexed detection capability of our QDs‐MSs@Ag array system, we analyzed nasal swab specimens of eight respiratory virus, including influenza A virus (FluA), influenza B virus (FluB), human parainfluenza virus type 1 (PIV1), human parainfluenza virus type 3 (PIV3), respiratory syncytial virus (RSV), adenovirus (HAD), human rhinovirus (HRV), and mycoplasma pneumoniae (MP). As shown in Figure 6A, different kinds of QDs‐MSs@Ag barcodes were functionalized with virus‐specific capture probes (Table S6). These barcodes were termed as QDs‐MSs@Ag^FluA‐CP^, QDs‐MSs@Ag^FluB‐CP^, QDs‐MSs@Ag^PIV1‐CP^, QDs‐MSs@Ag^PIV3‐CP^, QDs‐MSs@Ag^RSV‐CP^, QDs‐MSs@Ag^HAD‐CP^, QDs‐MSs@Ag^HRV‐CP^, and QDs‐MSs@Ag^MP‐CP^, respectively. Viral RNAs were extracted from patient samples using the MolPure Magnetic Universal Viral DNA/RNA Kit, which employs magnetic bead purification technology. The isolated RNAs were reverse‐transcribed to generate complementary DNA (cDNA), which subsequently underwent polymerase chain reaction (PCR) amplification using virus‐specific primers (Table S7). Notably, the 5’ end of each reverse primer was biotinylated to enable subsequent binding with streptavidin. After PCR, the amplified products were hybridized with eight barcodes. Following hybridization, the PE reporter was conjugated to the complex via the streptavidin‐biotin binding, and the multi‐channel decoding was executed via flow cytometry. The specific virus type is identified by the fluorescence emission of QDs‐MSs@Ag in their designated QD channels, while the viral load is quantified based on the PE channel signal intensity. To validate barcode specificity, we tested the system against all eight target respiratory viruses. As shown in Figure S40, high‐intensity PE signals with the MFI values over 1.1 × 10^4^ were detected only when QDs‐MSs@Ag barcodes hybridized with complementary viral RNA sequences, whereas mismatched viral RNA sequences yielded low PE signals. Moreover, the QDs‐MSs@Ag barcode system exhibited remarkable sensitivity, achieving uniform LOD values as low as 10 copies per reaction for all targets (Figure 6D). In contrast, the LOD values for detecting FluA and FluB acquired using QDs‐MSs were approximately 50 copies per reaction, demonstrating the excellent MEF enhancement from AgNC surface‐decoration (Figure S41). Building on these findings, we assessed the multiplexed biodetection performance of QDs‐MSs@Ag barcode system using 24 clinical patient samples. As shown in Figure 6E, all respiratory viruses present in each sample solution could be accurately detected, and the specific viral species could be determined simply by referring to the established barcodes‐MFI correlation.

## 3 Conclusions

In summary, this work demonstrates the controlled integration of densely‐immobilized NMNPs‐based hierarchical superstructures via precise engineering of polymer microsphere architecture and surface properties. The key innovation involves the synthesis and surface modulation of multifunctional polymer microspheres. Using the SPG membrane emulsification method, we successfully incorporated both magnetic and fluorescent nanoparticles within the microspheres. Subsequent deliberate removal of surfactants from the multifunctional polymer microspheres created a metastable surface state, triggering spontaneous and rapid co‐assembly with NMNPs to achieve on‐demand integration of plasmonic properties with other functionalities. Through systematic investigation of co‐assembly behaviors between various polymer microspheres and NMNPs, we established that the process is primarily driven by thermodynamically favorable heterocoagulation and coordination interactions.

Using PSMA microspheres as a model system, we characterized their physical properties before and after surfactant removal, demonstrating a decrease in the surface charges, an increase in the hydrated diameter, and an enhanced aggregation tendency upon surfactant elimination. When mixed with excess noble metal nanoparticles (e.g., Ag, Au) of various sizes, rapid spontaneous co‐assembly occurred within 1 min, with the surface loading coverage rate gradually increasing to about 46.5% and tending to equilibrium within 5 min. Semi‐quantitative analysis indicated maximum coverage rates stabilized near 50%, independent of microsphere or noble metal nanoparticle dimensions, confirming consistent surface properties.

We anticipate that these results are beneficial to multiple research fields. First, noble metal applications are often constrained by nanoparticle aggregation. Our method enables highly uniform NMNP distribution through a simple yet effective strategy, demonstrating exceptional catalytic performance in both organic transformations and enzyme‐mediated cascade reactions. Second, the developed microsphere system achieves homogeneous fluorescent nanoparticle encapsulation while maintaining optical properties. This strategy substantially enhances encoding capacity, showing particular promise for high‐throughput in vitro diagnostics with clinical samples. Finally, the successful assembly of plasmonic‐magnetic and plasmonic‐fluorescent hybrid systems establishes a versatile platform for constructing more complex hierarchies with integrated functionalities.

## 4 Experimental Section

### 4.1 Materials

Poly (styrene‐co‐maleic anhydride) (PSMA, *M*_W_ = 224 000, 7 wt.% anhydride), polyvinylpyrrolidone (PVP, *M*_W_ = 55 000), silver nitrate (AgNO_3_), NHydroxysulfosuccinimide sodium salt (NHS), N‐(3‐Dimethylaminopropyl)‐N’‐ethylcarbodiimide hydrochloride (EDC), ethylene glycol (EG), and 1,5‐pentanediol (PD) were purchased from Sigma. Sodium sulfide nonahydrate (Na_2_S·9H_2_O), sodium borohydride (NaBH_4_), gold acid chloride trihydrate (HAuCl_4_·3H_2_O), thioctic acid (TA), trichloromethane, alcohol, toluene, sodium dodecyl sulfate (SDS), hydrochloric acid (HCl), p‐Nitrophenol (4‐NP), methylene blue (MB), N, N‐dimethylformamide (DMF), glucose, hydrogen peroxide (H_2_O_2_, 30%) and glucose oxidase (GOx) were purchased from Aladdin. Sodium acetate solution (NaAc, pH = 5.2, 3 M) was purchased from Beyotime. Tris‐(2‐carboxyethyl)‐phosphine hydrochloride (TCEP), double‐distilled water (ddH_2_O), 10% SDS solution, and hybridization cocktails III (Contains 6 x SSC, 0.5% SDS, 100 µg mL^−1^ Salmon sperm DNA) were purchased from Sangon Biotech. PBST (1 M, pH = 7.4), MES buffer (0.1 M, pH = 4.7), and Na‐Ac buffer (0.1 M, pH = 4.0) were purchased from Shanghai Yuanye Bio‐Technology. Streptavidin‐R‐phycoerythrin (SAPE) and 96‐well polystyrene microtiter plates were purchased from ThermoFisher Scientific. 10 nm Fe_3_O_4_ nanoparticles were purchased from Zhejiang Orient Gene Biotech Co., Ltd. DNA strands were synthesized and HPLC‐purified by Sangon Biotech. MolPure Magnetic Universal Viral DNA/RNA Kit was purchased from Yeasen Biotech Co., Ltd. Ultrapure water (18.2 MΩ cm) was obtained with a Millipore Milli‐Q system. All reagents were of analytical grade and used without any purification.

### 4.2 Characterizations

SEM images and energy‐dispersive spectrometer (EDS) mapping were captured using a scanning electron microscope (Carl Zeiss, Gemini 300, Germany). TEM images were obtained by a 120 kV biology transmission electron microscope (FEI, Tecnai G2 SpiritBiotwin, USA). Dynamic light scattering (DLS) and Zeta potential were analyzed with Zetasizer Nano S (omni, Brookhaven). QDs‐MSs@Ag array and samples of multiplexed detection were analyzed by a flow cytometer (Beckman Coulter, CytoFlex, USA). Absorption spectra were measured by a UV‐2550 Shimadzu UV–vis spectrophotometer. The coverage rate was semi‐quantitatively determined by ImageJ.

### 4.3 Preparation of PSMA MSs via SPG Membrane Emulsification

A SPG glass membrane (with pore sizes of 3, 5, 8, 10, or 15 µm, SPG Technology, Japan) was installed into an emulsification device equipped with an external pressure‐type micro‐kit (SPG Technology, Japan). The oil phase was prepared by dissolving 125 mg PSMA in 2 mL of toluene. The aqueous continuous phase contained 5 mg mL^−1^ SDS as stabilizer. After emulsion formation, the mixture was stirred for 24 h to complete solvent evaporation within the droplets. PSMA MSs were collected by centrifugation (6500 rpm, 3 min), followed by three washes with ultrapure water. The preparation of other polymer MSs followed an identical protocol to the PSMA MSs.

### 4.4 Synthesis of AgNCs

AgNCs were prepared using the method reported previously [36]. Briefly, EG served as the solvent and AgNO_3_ as the precursor. A 30 mL aliquot of EG was heated to 155 °C in an oil bath under magnetic stirring for 1 h. After sequential addition of 0.35 mL Na_2_S·9H_2_O (3 mM in EG) and 7.5 mL PVP (20 mg mL^−1^ in EG), 2.5 mL AgNO_3_ (48 mg mL^−1^ in EG) was injected into the mixture. Stirring continued until a ruddy‐brown colloidal suspension formed. The reaction was quenched by immersion in an ice‐water bath. The product of 48.3 nm‐AgNCs was diluted with acetone, collected by centrifugation (10 000 rpm, 5 min), washed with ultrapure water three times, and resuspended in 12 mL of ultrapure water for subsequent use. AgNCs of defined sizes were synthesized by varying AgNO_3_ volumes. 25.4 nm‐, 32.6 nm‐, and 72.0 nm‐AgNCs were obtained using 1.2, 1.25, and 3.75 mL of AgNO_3_ solution, respectively.

### 4.5 Synthesis of Au Nanoparticles

0.15 mL of AgNO_3_ (0.02 M in PD) was added to 5.0 mL of boiling PD. Subsequently, 3.0 mL of PVP (0.15 M in PD) and 3.0 mL of HAuCl_4_·3H_2_O (0.05 M in PD) solutions were alternately added every 30 s over 7.5 min. The mixture was then refluxed for 1 h. After cooling to room temperature, large aggregates were removed by centrifugation at 500 rpm for 5 min [67]. The product was purified through three cycles of dispersion/precipitation in ethanol to remove excess PVP, and finally dispersed in 10 mL ultrapure water.

### 4.6 Synthesis of Au Nanostars

Au nanostars were synthesized via a seed‐mediated growth method in previous work [68]. Gold seed solution was prepared by diluting 1 mL of 1% w/v HAuCl_4_·3H_2_O aqueous solution to 90 mL with ultrapure water, adding 2 mL of 38.8 mM sodium citrate, and slowly introducing 1 mL of freshly prepared NaBH_4_ solution (0.075% w/v in 38.8 mM sodium citrate). This mixture was stirred overnight at room temperature. Subsequently, 5.0 g of PVP was dissolved in 50 mL of the seed solution and stirred for 24 h to yield PVP‐coated seeds. For nanostar growth, 1.5 g PVP was dissolved in 15 mL DMF, followed by the addition of 82 µL 50 mM HAuCl_4_·3H_2_O aqueous solution and 0.1 mL PVP‐coated seed solution. After stirring for 3 h, the product was centrifuged and washed twice with ethanol and ultrapure water, respectively. The pellets were redispersed in 1 mL of ultrapure water for further use.

### 4.7 Synthesis of Au‐Ag Nanocages

Typically, 100 µL of the previously stored 48.3 nm‐AgNCs was suspended in 5 mL of 9 mM PVP solution within a 50 mL round‐bottom flask under magnetic stirring [69]. The mixture was heated to boiling and maintained for 10 min. After that, 4 mL of an aqueous 0.1 mM HAuCl_4_ solution was infused into the flask using a syringe pump at a constant rate of 0.75 mL min^−1^. Heating and stirring continued for an additional 10 min until the solution color stabilized. Subsequently, the reaction mixture was cooled to room temperature. The resulting Au‐Ag nanocages were isolated by centrifugation, washed once with saturated NaCl solution to remove AgCl, and then rinsed several times with water to eliminate PVP and NaCl. Finally, the purified Au‐Ag nanocages were redispersed in ultrapure water and stored in the dark at 4 °C until use.

### 4.8 Co‐Assembly of PSMA MSs and AgNCs

Typically, a mixture containing 100 µL PSMA MSs (6.4 µm, 4 mg mL^−1^) and 20 µL 48.3 nm‐AgNCs (0.64 mg mL^−1^) was prepared in a 2.0 mL centrifuge tube. Ultrapure water was added to adjust the total volume to 200 µL. The mixture was immediately homogenized by ultrasonication for 5 min. Then, residual free Ag NCs were removed by filtration with the membrane (pore size, 1.2 µm) and washed with water three times to obtain the co‐assembly of MSs@Ag. The mixing mass ratio of PSMA MSs and AgNCs was varied by adjusting Ag NC volume while maintaining a constant PSMA concentration. The co‐assembly of PSMA MSs@Au nanoparticles, PSMA MSs@Au nanostars, and PSMA MSs@Au‐Ag nanocages was conducted following the same protocol.

### 4.9 Preparation of Magnetic MSs@Ag

The procedure was identical to the PSMA MSs@AgNCs protocol, except for the oil phase composition during SPG membrane emulsification: 125 mg PSMA and 5.0 mg 10 nm Fe_3_O_4_ nanoparticles were co‐dispersed in toluene.

### 4.10 4‐NP Reduction

Catalytic activity of mag‐MSs@Ag was quantified by UV‐vis spectrophotometry. A reaction mixture containing 1.0 mL 4‐NP (0.3 mM) and 0.9 mL NaBH_4_ (0.3 M) was prepared in a quartz cuvette. The reaction was initiated by adding 0.1 mL mag‐MSs@Ag suspension (1.5 mg catalyst). Negative controls omitted the catalyst addition. The absorbances of the reaction were recorded by UV spectroscopy at different reaction times. When the reaction was accomplished, mag‐MSs@Ag were collected from the mixture by a magnet. To estimate the catalytic activity of this catalyst after the reduction reaction, the recycled mag‐MSs@Ag was reused.

### 4.11 MB Reduction

A suspension of magnetic MSs@Ag (1.5 mg in 0.1 mL H_2_O) was mixed with 1.5 mL MB (0.03 mM). The reaction was initiated by adding 0.4 mL NaBH_4_ (60 mM). Absorbance decay at 664 nm was monitored at 30 s intervals. After the reaction, the catalysts were magnetically recovered and reused.

### 4.12 Preparation of mag‐MSs@Ag‐GOx

0.1 mL mag‐MSs@Ag (30 mg mL^−1^) were first functionalized by pH adjustment to 11.0 with 7.5 µL NaOH (0.5 M), followed by the addition of 0.5 mL thioctic acid (TA, 15 mM in ethanol). The mixture was vortexed at 1000 rpm for 2 h, centrifuged, and redispersed in 0.5 mL ultrapure water to yield mag‐MSs@Ag‐TA. For enzyme conjugation, mag‐MSs@Ag‐TA was activated with 10 µL NHS (20 mg mL^−1^) and 10 µL EDC (30 mg mL^−1^) for 30 min. Glucose oxidase (GOx, 5.6 mg) in 1 mL water was then added, and the reaction proceeded under stirring for 1 h. The product was collected by centrifugation, washed three times, and finally dispersed in 0.3 mL ultrapure water.

### 4.13 Peroxidase‐Mimetic and Cascade Reaction Assays

Peroxidase catalytic activity of mag‐MSs@Ag was measured by adding 50 µL suspension (10 mg mL^−1^) to sodium acetate (Na‐Ac) buffer solution (0.1 M, pH = 4.0) containing TMB (10 mM) and H_2_O_2_ (10 mM). The absorbance at 370 and 652 nm was measured by UV–vis spectroscopy. For enzyme‐nanozyme cascade reactions, mag‐MSs@Ag‐GOx composites (50 µL, 10 mg mL^−1^) were incubated with 0.6 mL glucose (0.5 M), 0.2 mL TMB (10 mM), and 1.15 mL Na‐Ac buffer (0.1 M, pH = 4.0). The resulting solution was tested by UV–vis spectroscopy.

### 4.14 Evaluation of MEF Effect

Thiolated ssDNA capture probes (300 pmol, 18 bp‐ssDNA, 45 bp‐ssDNA, 84 bp‐ssDNA) were reduced in 400 µL TCEP (10 mM) for 2 h, precipitated with 150 µL NaAc (3 M, pH 5.2) and 1.1 mL ice‐cold ethanol (−80 °C, 30 min), then centrifuged (12 000 rpm, 10 min). For conjugation to PSMA MSs@AgNCs, 1 × 10^6^ MSs were incubated with 100 pmol of the reduced thiolated ssDNA in 100 µL PBST containing 1.77 mg NaCl for 2 h, followed by sequential washing with PBST and 0.1% SDS. The conjugated MSs were finally resuspended in 120 µL ddH_2_O for later use. For hybridization, 6 µL of the MSs suspension was combined with 2 µL biotin‐modified complementary ssDNA (concentration gradient: 0.001–100 nM) and 12 µL hybridization cocktail III and incubated at 45 °C for 30 min. Unhybridized DNA was removed by washing with PBST. 100 µL of SAPE (4 µg mL^−1^) was added and incubated in the dark with oscillation for 15 min. Unbound SAPE was removed by three washes with PBST. The MSs were then resuspended in PBST and analyzed by flow cytometry, and the average PE fluorescence intensity was recorded. For control experiments using plain PSMA MSs, an equivalent number of MSs were incubated with 100 pmol aminated capture probe and 2 mg EDC in 95 µL MES buffer for 30 min, followed by the addition of 5 µL EDC (10 mg mL^−1^ in MES) and a further 30 min incubation under rotation. The subsequent hybridization and detection procedures were identical.

### 4.15 Preparation of QDs‐MSs@Ag Array

The procedure was identical to the PSMA MSs@AgNCs protocol, except for the oil phase composition during SPG membrane emulsification. Specifically, 125 mg PSMA and different amounts of QDs were co‐dispersed in toluene. The mass of each QD type added was as follows: 0, 0.05, 0.20, 1.0, and 2.0 mg of QD_512_, 0, 0.075, 0.25, 1.25, and 2.5 mg of QD_628_, 0, 0.1, 0.5, 2.0, and 4.0 mg of QD_716_.

### 4.16 QDs‐MSs@Ag Barcodes for Respiratory Virus Detection

*V*iral RNAs were isolated from nasal swab samples utilizing the MolPure Magnetic Universal Viral DNA/RNA Kit, which incorporates magnetic bead purification technology. The extracted RNAs were then subjected to reverse transcription to synthesize cDNA, which was subsequently amplified via PCR using specific primers and DNA polymerase. PCR reactions (20 µL) contained 2 µL cDNA template, 0.8 µL DNA polymerase, 0.5 µL each of forward and reverse primers, and ddH_2_O to a final volume of 20 µL. The reaction conditions are: pre‐denaturation at 95 °C for 5 min and denaturation at 95 °C for 30 s, annealing at 56 °C for 30 s, extension at 72 °C for 30 s, 35 cycles. Samples were then held for final extension at 72 °C for 5 min and kept at 4 °C. Eight distinct QDs‐MSs@Ag barcodes (QDs‐MSs@Ag^FluA‐CP^, QDs‐MSs@Ag^FluB‐CP^, QDs‐MSs@Ag^PIV1‐CP^, QDs‐MSs@Ag^PIV3‐CP^, QDs‐MSs@Ag^RSV‐CP^, QDs‐MSs@Ag^HAD‐CP^, QDs‐MSs@Ag^HRV‐CP^, and QDs‐MSs@Ag^MP‐CP^) were prepared by labeling with 100 pmol of their respective thiolated capture probes (containing oligonucleotide sequences complementary to partial target sequences) and subsequently mixed in equal proportions. For hybridization, 5 µL of PCR product was denatured at 95 °C for 5 min, combined with 6 µL of QDs‐MSs@Ag barcodes suspension and 9 µL of hybridization cocktail III, then incubated at 45 °C for 30 min. The mixture was washed three times with PBST and incubated with 100 µL SAPE (4 µg mL^−1^) for 15 min. After three washes with PBST to remove unbound SAPE, the MSs were resuspended in 100 µL PBST and analyzed by flow cytometry.

### 4.17 Statistical Analysis

All data were presented as the means ± standard deviation (SD). Statistical analyses were performed using the Origin software (version 2025) and ImageJ.

## Funding

This work was financially supported by the National Natural Science Foundation of China, Project No. 82372089 (W. L.), 82272823 (X.Y.). Inner Mongolia Autonomous Region‐Shanghai Jiao Tong University Science and Technology Cooperation Special Project, Project No. KJXM2023‐02‐01, KJXMZ20251104 (W.L.). Natural Science Foundation of Shanghai, Project No. 25CL2900900 (W.L.), 24141900402 (W. L), 23ZR1434600, and 25CL2900902 (X.Y.).

## Conflicts of Interest

The authors declare no conflicts of interest.

## Supporting information




**Supporting File 1**: advs73439‐sup‐0001‐SuppMat.pdf.

**Supporting File 2**: advs73439‐sup‐0002‐MovieS1.mp4.

## Data Availability

The data that support the findings of this study are available in the supplementary material of this article.
