# Supplementary material for: Ultrafast Dense Immobilization of Noble Metal Nanoparticles on Customizable Multifunctional Polymer Microspheres for Heterogeneous Catalysis and Multiplexed Biodetection
Source: Adv Sci (Weinh). 2025 Dec 20;13(17):e20357. doi: 10.1002/advs.202520357 (PMC13042535; doi:10.1002/advs.202520357)
Supplement: Supplementary file 1 — Supporting File 1: advs73439‐sup‐0001‐SuppMat.pdf. [file ADVS-13-e20357-s001.docx]

Supporting Information

Ultrafast Dense Immobilization of Noble Metal Nanoparticles on Customizable Multifunctional Polymer Microspheres for Heterogeneous Catalysis and Multiplexed Biodetection

Jie Zhao, Xinyi Liu, Dan Li, Xuefei Jing, Weijie Wu, Liangrui He, Yizhang Tang, Zhen Liu, Yulin Zeng, Haitian Sha, Huibin Qiu, Xujiang Yu*, Wanwan Li*

J. Zhao, X. Liu, D. Li, X. Jing, W. Wu, L. He, Y. Tang, Y. Zeng, H. Sha, X Yu, W. Li

State Key Lab of Metal Matrix Composites, School of Materials Science and Engineering, Zhangjiang Institute for Advanced Study, Shanghai Jiao Tong University, 800 Dongchuan Road, Shanghai 200240, P. R. China

E-mail: [yuxuj1017@sjtu.edu.cn](mailto:yuxuj1017@sjtu.edu.cn); [wwli@sjtu.edu.cn](mailto:wwli@sjtu.edu.cn)

Z. Liu

School of Materials Science and Engineering, Nanjing University of Science and Technology, Nanjing, Jiangsu 210094, P. R. China

H. Qiu

School of Chemistry and Chemical Engineering, Zhangjiang Institute for Advanced Study, Frontiers Science Center for Transformative Molecules, State Key Laboratory of Metal Matrix Composites, Shanghai Jiao Tong University, Shanghai 200240, P. R. China

W. Li

Inner Mongolia Research Institute of Shanghai Jiao Tong University, Huhehot, 010052, P. R. China

Contents:

Figure S1 to S41

Tables S1 to S7

Legend for Movie S1

Other Supporting Information Movies S1 was provided separately

**Figure S1.** Chemical structure of PSMA.


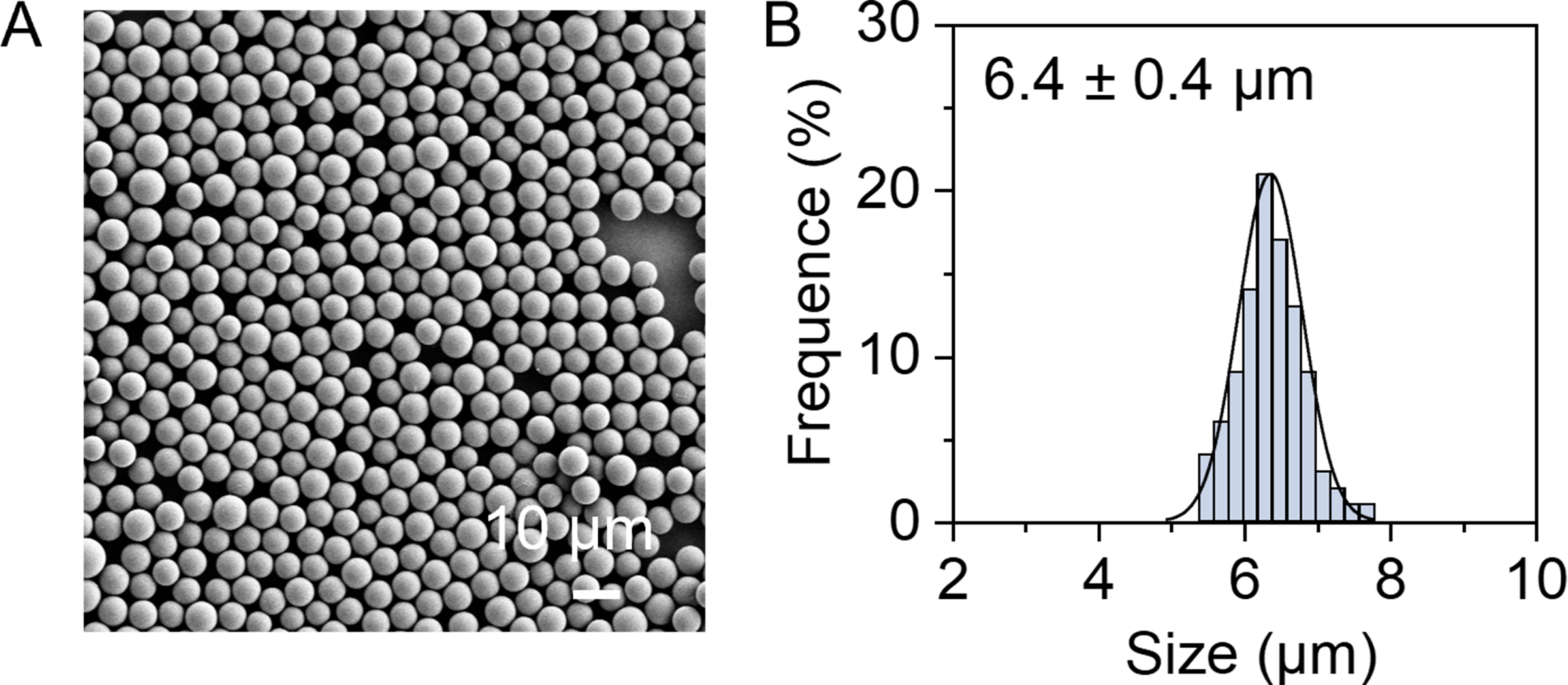


**Figure S2.** SEM image (A) and corresponding size distribution histogram (B) of PSMA MSs. Mean particle diameter: 6.4 ± 0.4 μm.


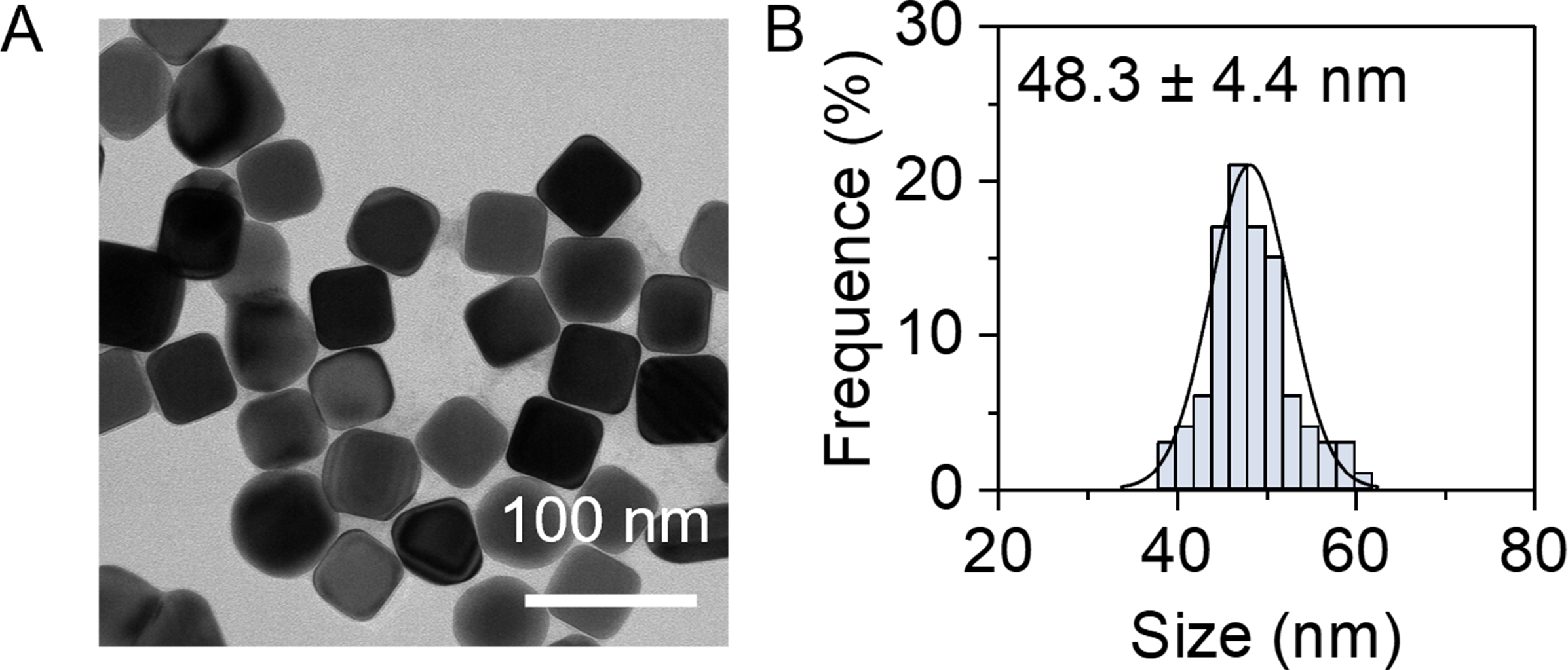


**Figure S3.** (A) TEM image of AgNCs. (B) Particle-size distribution histogram. Mean particle diameter: 48.3 ± 4.4 nm.


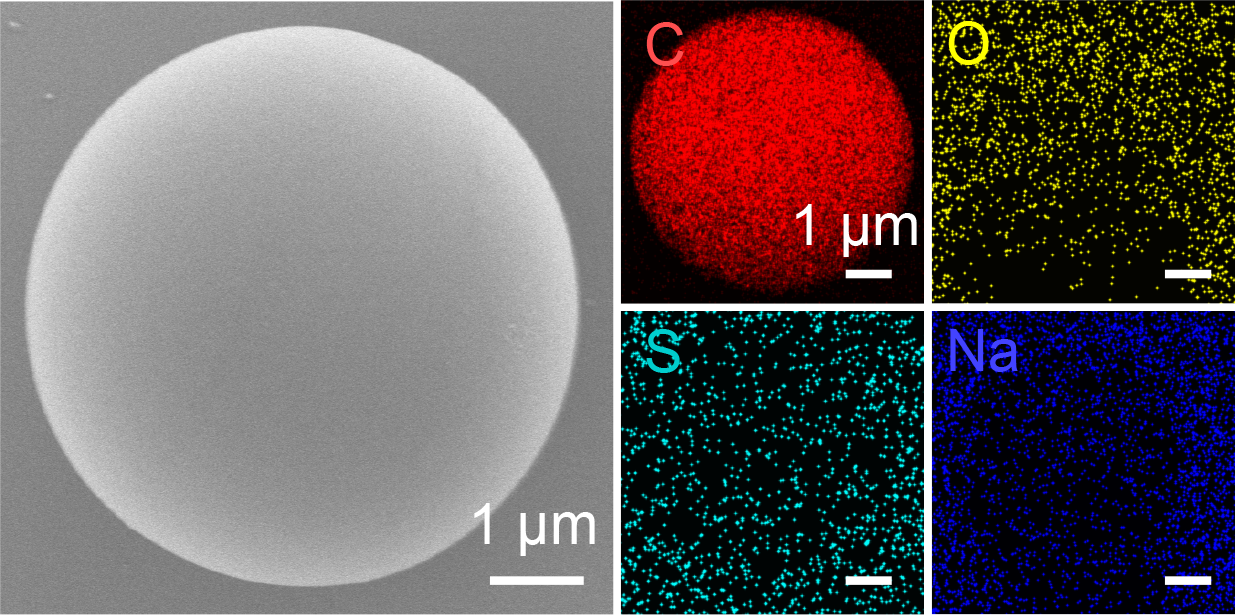


**Figure S4.** SEM image of SDS-free PSMA MSs and the corresponding EDS mapping. EDS analysis confirmed complete removal of SDS surfactant (absence of detectable Na and S signals). C and O signals originate from the PSMA polymer backbone. (Accelerating voltage: 15 kV, working distance: 8 mm).


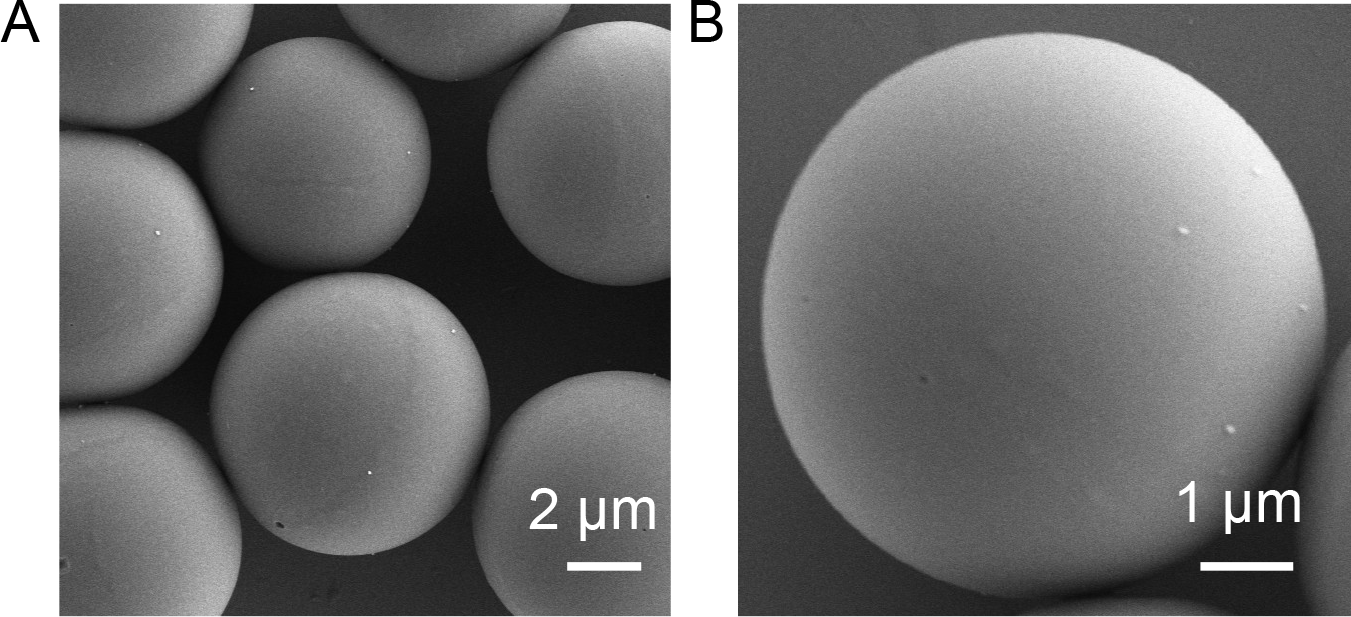


**Figure S5.** SEM analysis of attempted co-assembly between SDS-stabilized PSMA MSs and AgNCs. (A) Low-magnification overview. (B) High-magnification image of representative SDS-stabilized PSMA MSs. The SEM images reveal that the SDS-stabilized PSMA MSs appear uncoated, with minimal to no evidence of co-assembled AgNCs on their surfaces.


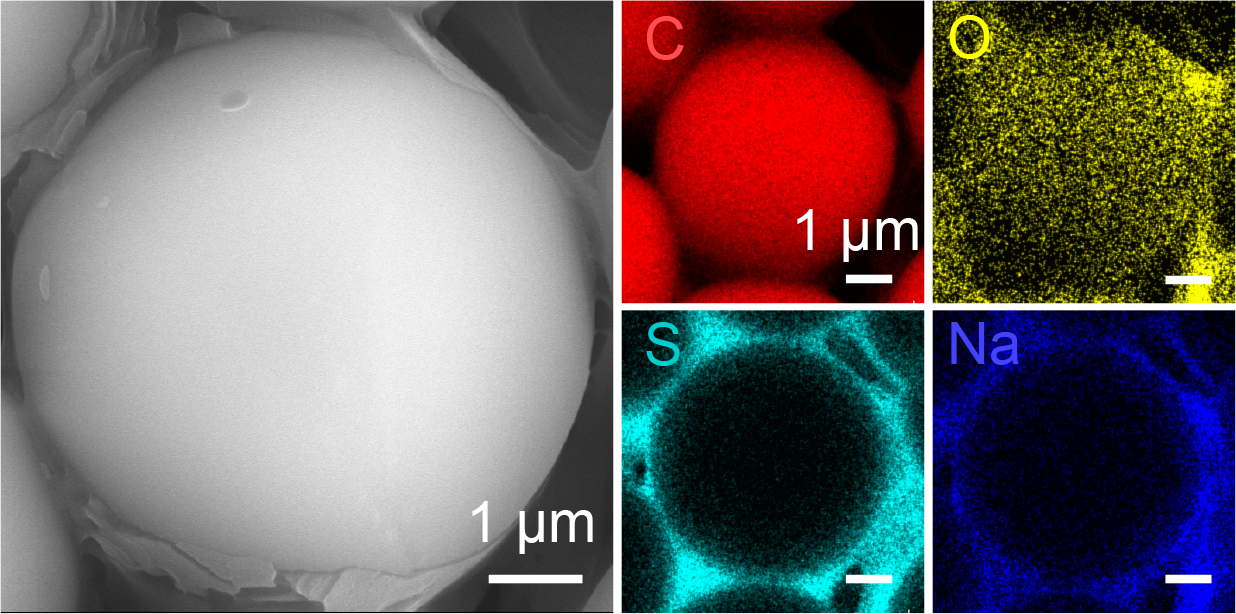


**Figure S6.** SEM image of SDS-stabilized PSMA MSs and the corresponding EDS mapping. EDS analysis confirms SDS surfactant functionalization through the detection of characteristic Na and S signals.


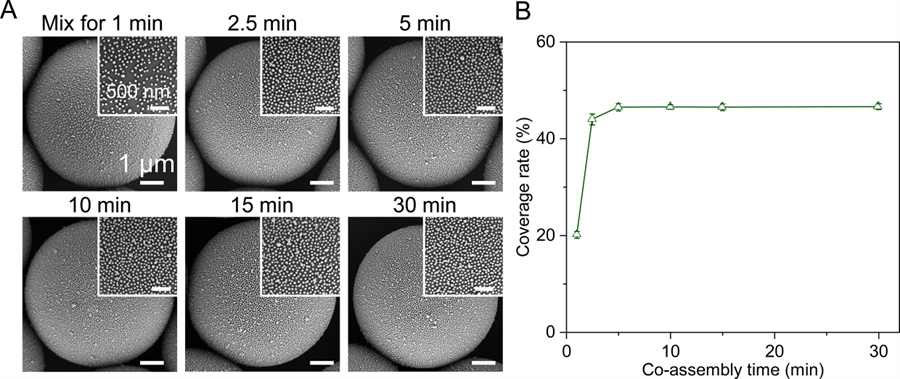


**Figure S7.** (A) SEM images and (B) coverage rates of the co-assembly of PSMA MSs and AgNCs at different reaction time. Co-assembly occurred rapidly within 1 min after mixing PSMA MSs and AgNCs at a mass ratio of 31.3:1. The amount of AgNCs co-assembled onto PSMA MSs reached equilibrium within approximately 5 min, achieving 46.5% coverage, with no significant changes observed even after extending the mixing time to 30 min.

**
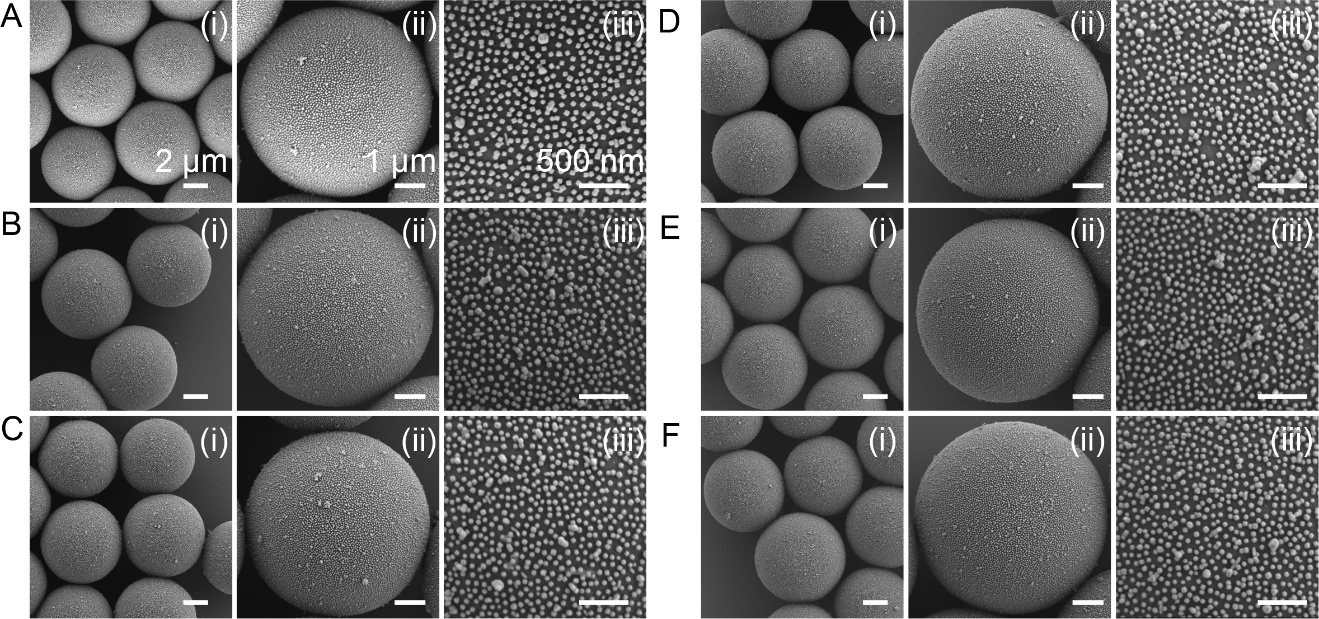
**

**Figure S8.** The stability of co-assembled PSMA MSs@Ag after 24-hour incubation at different temperatures. (A) 4 °C, (B) 25 °C, (C) 37 °C, (D) 50 °C, (E) 60 °C and (F) 80 °C. The core-shell architectures maintained structural integrity, demonstrating exceptional thermal stability of PSMA MSs@Ag.


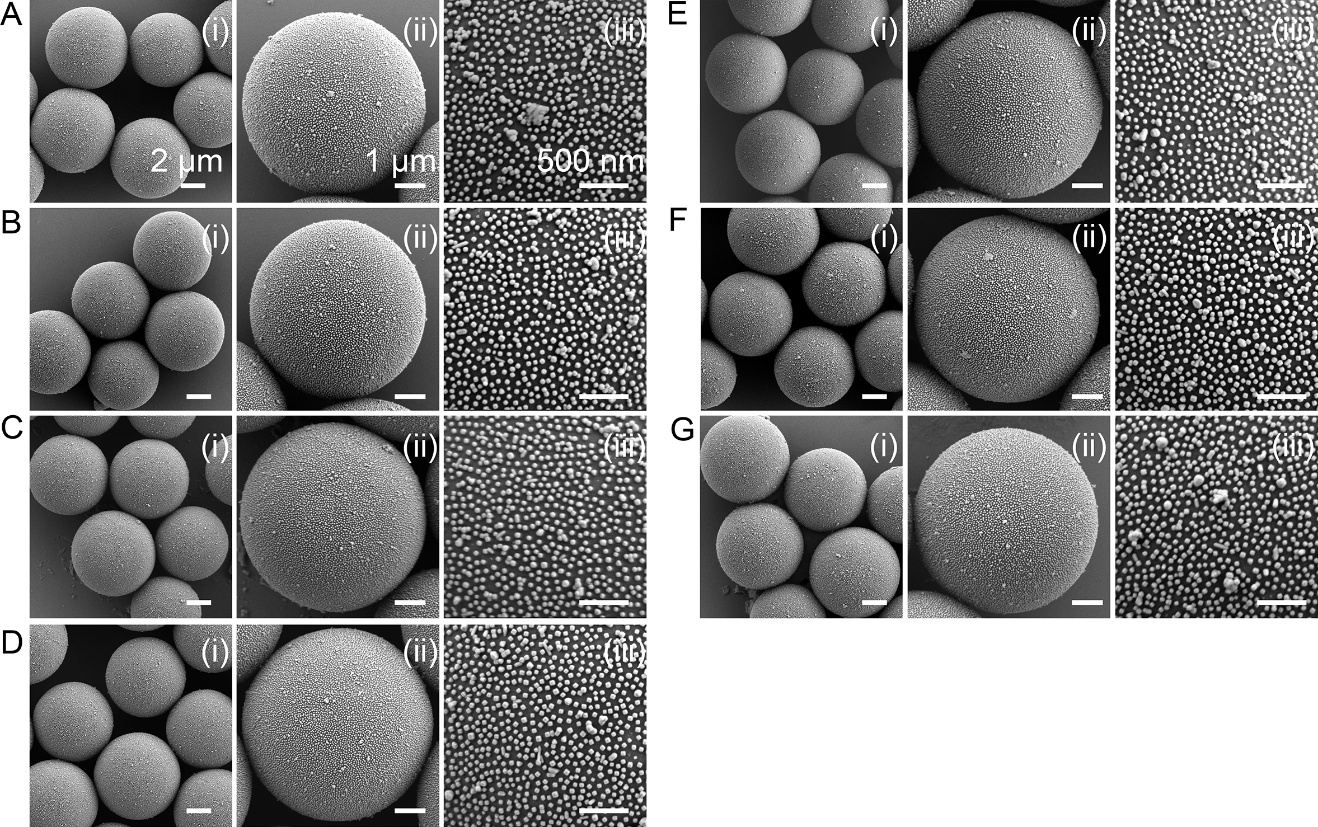


**Figure S9.** The stability of co-assembled PSMA MSs@Ag under different pH. (A) pH = 1, (B) pH = 3, (C) pH = 5, (D) pH = 7, (E) pH = 9, (F) pH = 11, and (G) pH = 13. The architecture maintained structural integrity, demonstrating exceptional pH stability.


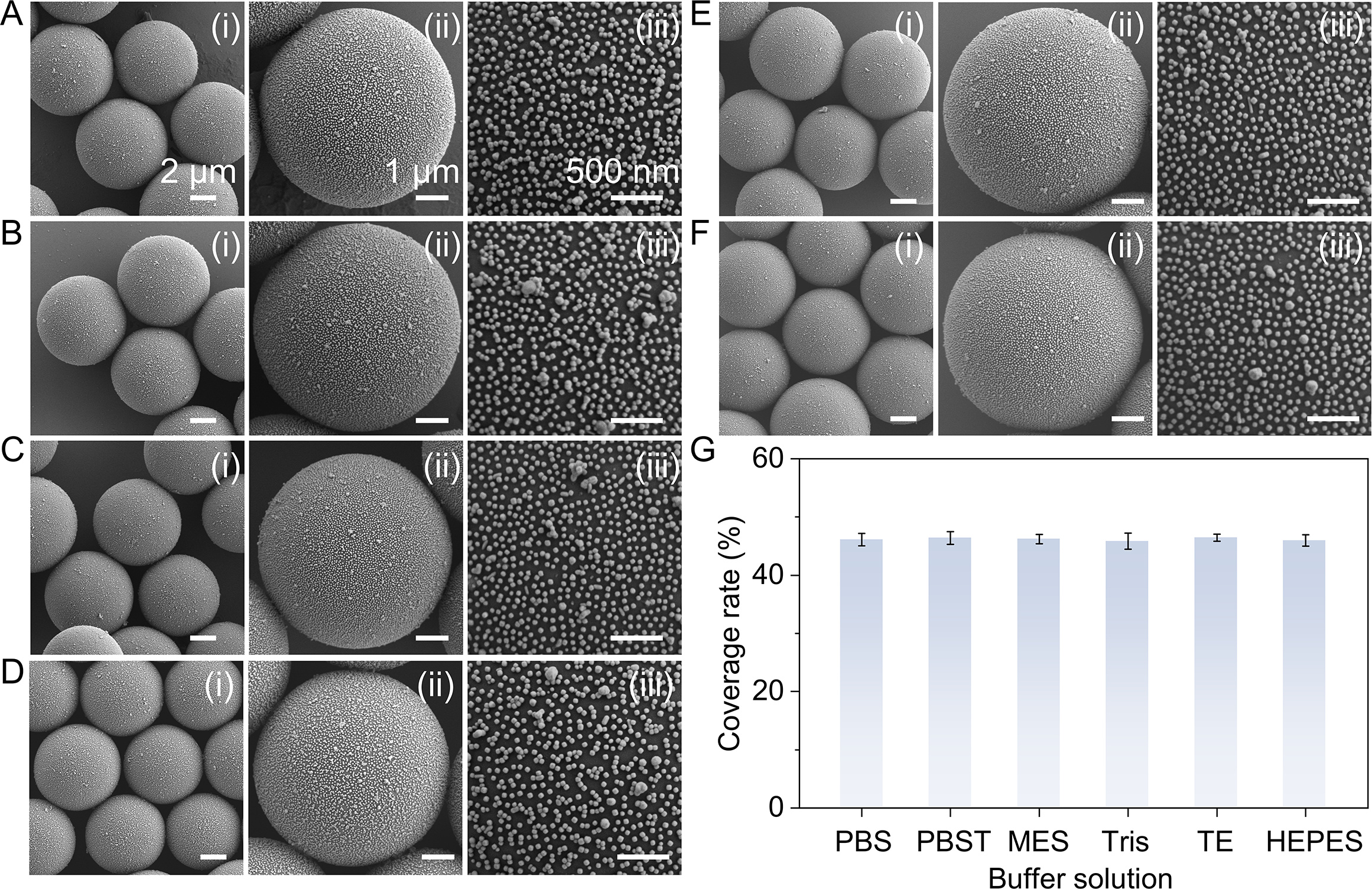


**Figure S10.** Buffer solution stability of co-assembled PSMA MSs@Ag. (A) PBS, (B) PBST, (C) MES, (D) Tris, (E) TE, and (F) HEPES. (G) The coverage rates of different buffer solutions. The hybrid structures exhibited remarkable colloidal stability and retained their structural integrity across the tested buffer systems, thereby highlighting their outstanding compatibility.


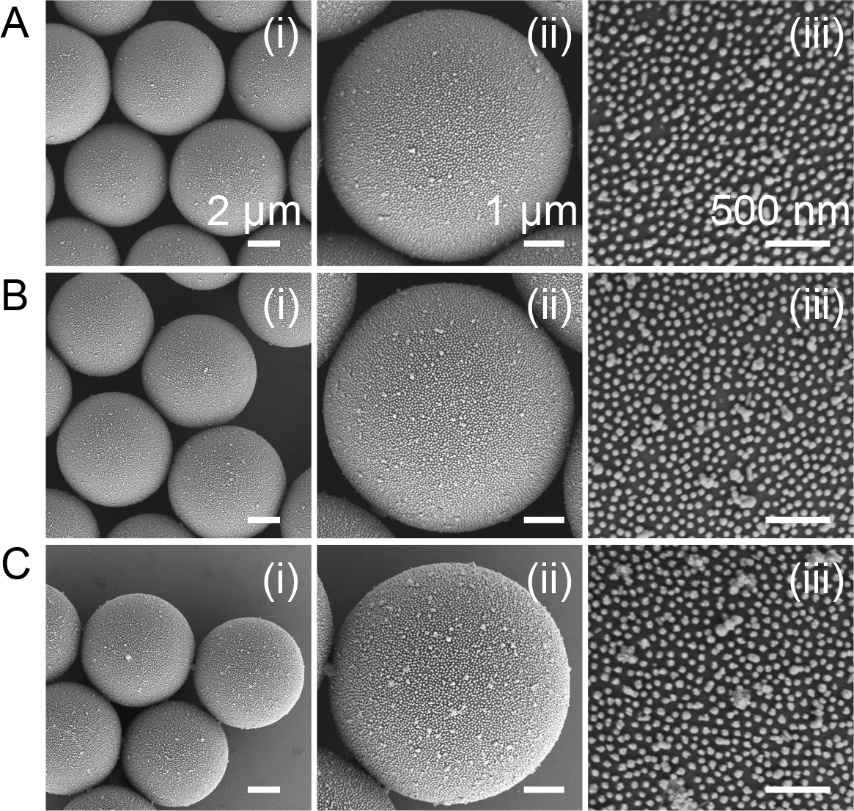


**Figure S11.** The ultrasonic stability of co-assembled PSMA MSs@Ag over different time. (A) 10 min, (B) 20 min, and (C) 30 min. The structures maintained their structural integrity, exhibiting no signs of fragmentation or dissociation of components throughout the entire ultrasonic treatment, thereby underscoring their remarkable resilience to ultrasonics.


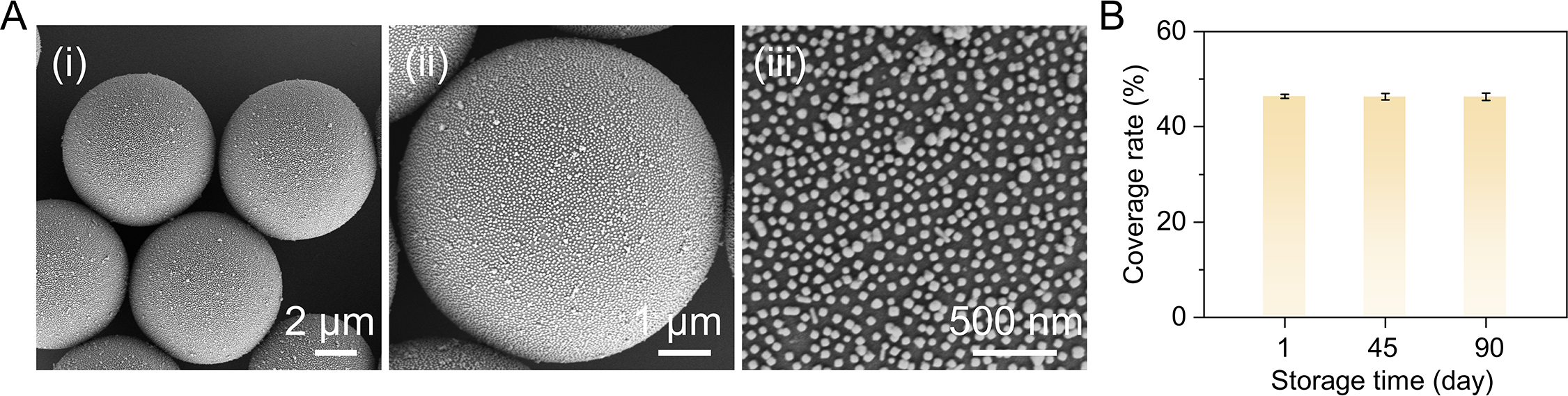


**Figure S12.** (A) SEM images of PSMA MSs@Ag after 90-day storage at 4 °C. (B) The coverage rates under different storage time. The architecture maintained structural integrity, confirming exceptional long-term storage stability.


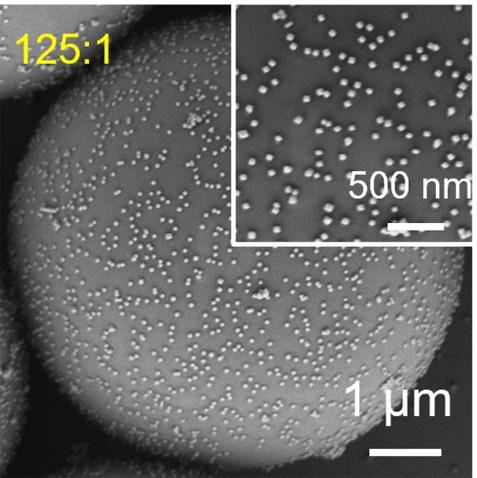


**Figure S13.** SEM images of PSMA MSs@Ag co-assembled at a PSMA MSs to AgNCs mass ratio of 125:1. The coverage rate was calculated to be 9.5%.


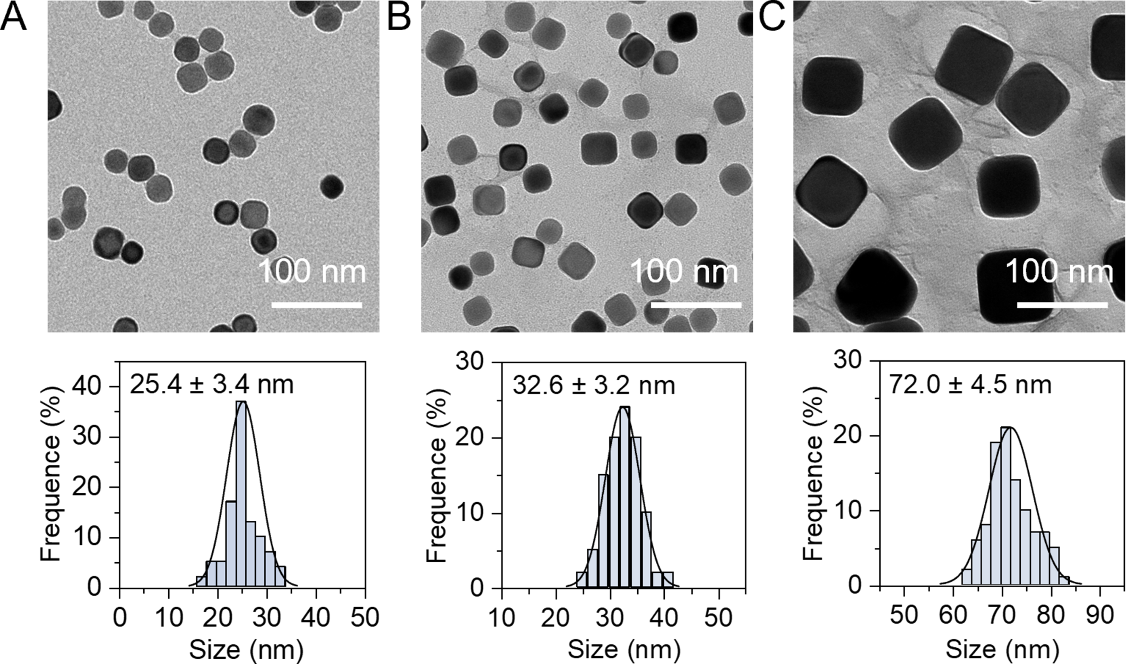


**Figure S14.** TEM images and particle-size distributions of AgNCs. (A) 25.4 nm AgNCs. (B) 32.6 nm AgNCs and (C) 72.0 nm AgNCs. Scale bar: 100 nm.


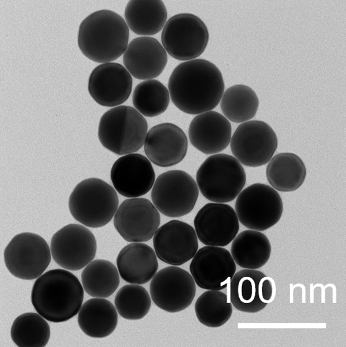


**Figure S15.** TEM image of synthesized Au nanoparticles with an average diameter of ~ 50 nm. Scale bar: 100 nm.


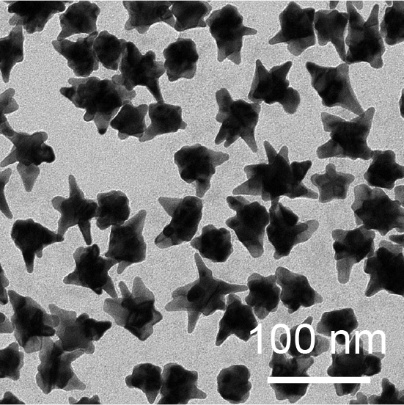


**Figure S16.** TEM image of synthesized Au nanostars with an average diameter of ~ 45 nm. Scale bar: 100 nm.


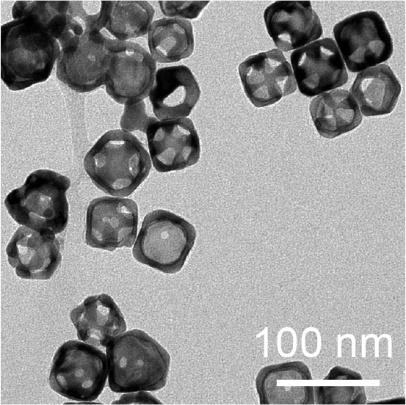


**Figure S17.** TEM image of synthesized Au-Ag nanocages with an average diameter of ~ 48 nm. Scale bar: 100 nm.


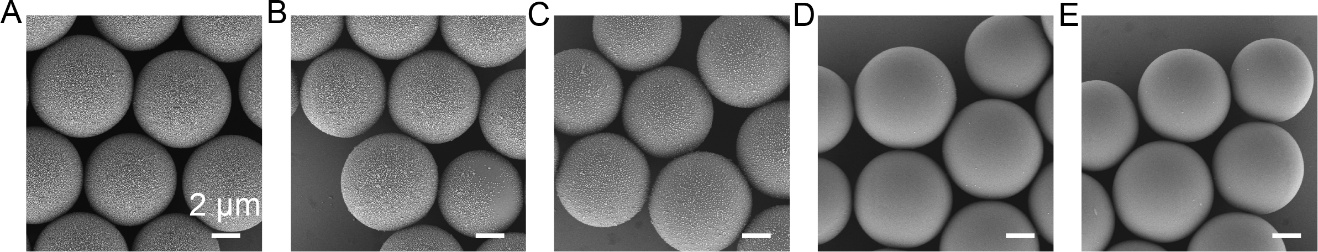


**Figure S18.** Effect of additional SDS on co-assembly of PSMA MSs and AgNCs. SDS concentrations: (A) 0 μM, (B) 1.5 μM, (C) 7.5 μM, (D) 15 μM, (E) 150 μM. The coverage decreased with increasing SDS concentration, showing a sharp reduction at 15 μM.


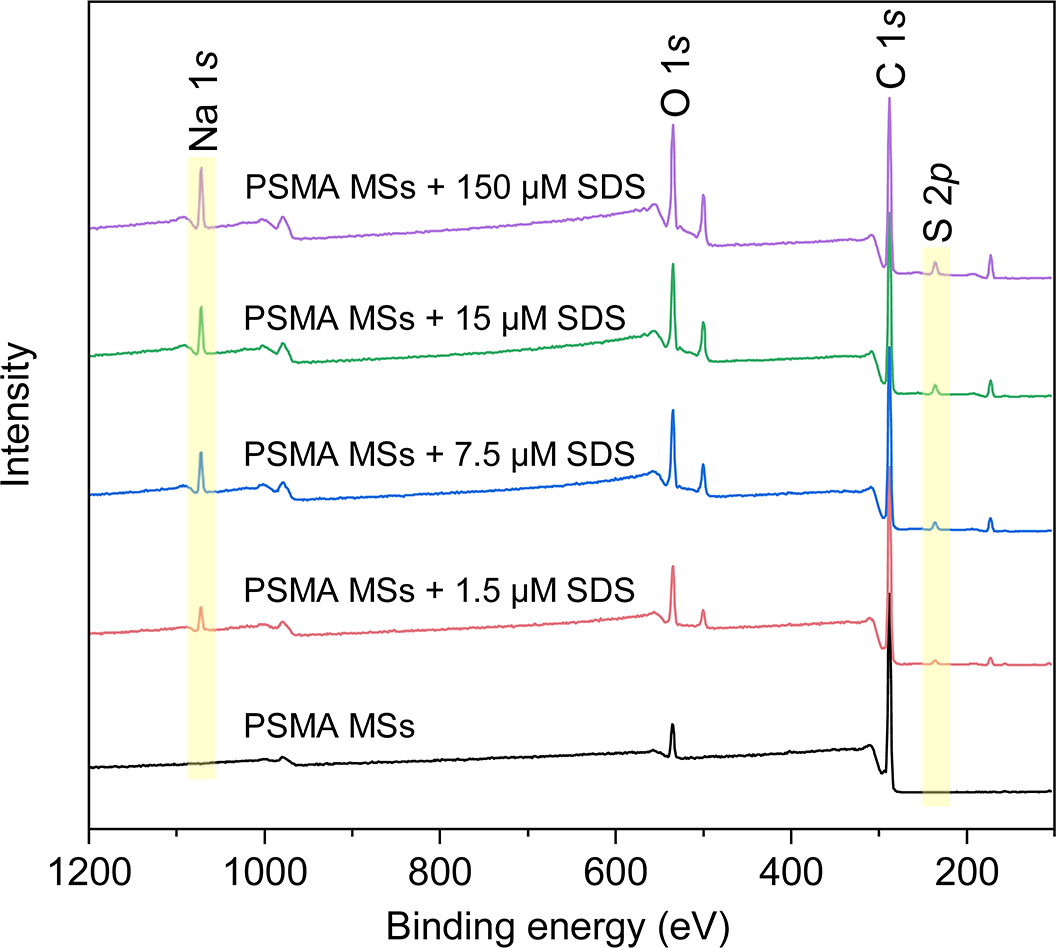


**Figure S19.** XPS spectra of pristine PSMA MSs and PSMA MSs with different amount of extra SDS. The appearance of characteristic Na and S signals confirmed the adsorption of SDS onto the metastable PSMA MS surface.


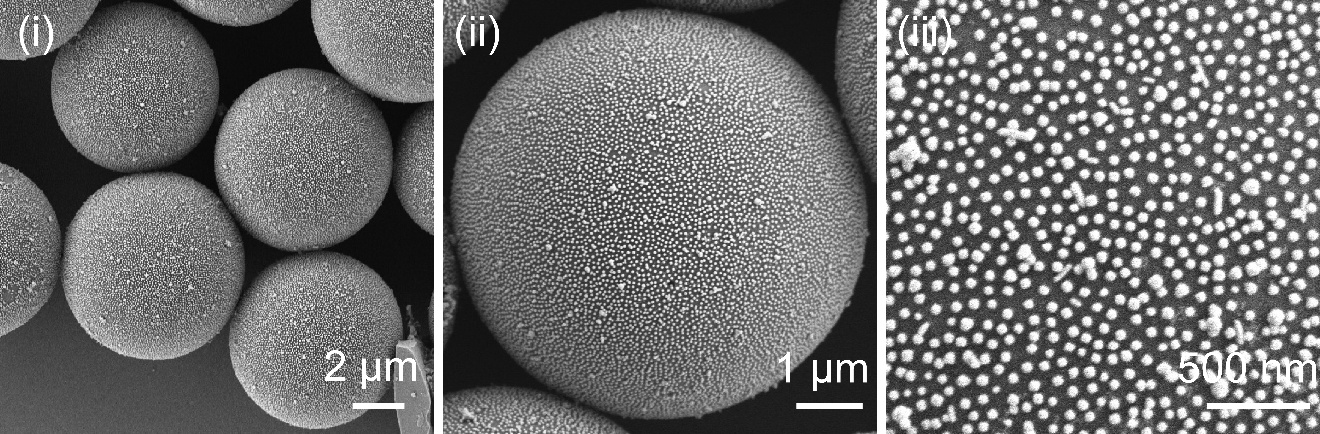


**Figure S20.** Stability of co-assembled PSMA MSs@Ag in SDS solution. The co-assembled structures remain stable after 24 h incubation.


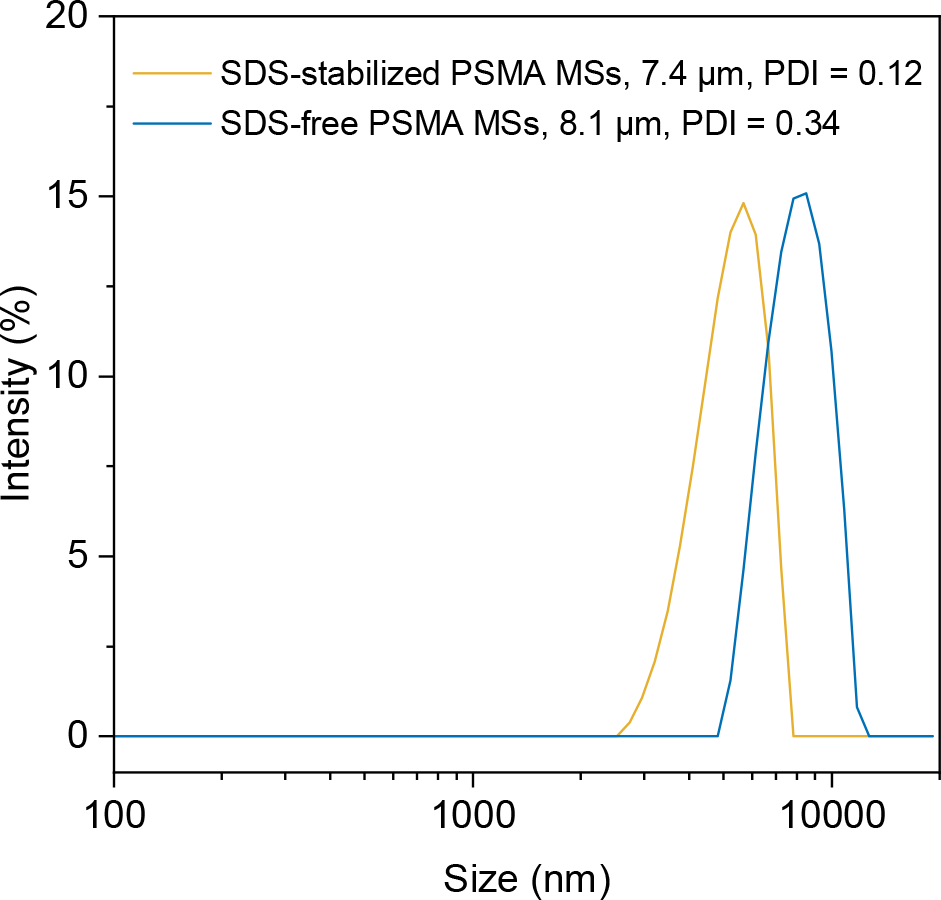


**Figure S21.** DLS analysis of the hydrated diameters of SDS-stabilized PSMA MSs and SDS-free PSMA MSs. The DLS measurements revealed that SDS-stabilized PSMA MSs exhibited a mean hydrated diameter of 7.4 μm, accompanied by a polydispersity index (PDI) of 0.12, indicating a relatively uniform and narrow size distribution. In contrast, SDS-free PSMA MSs stabilization displayed a mean hydrated diameter of 8.1 μm, coupled with a notably higher PDI of 0.34, which not only suggests a wider dispersion in particle sizes but also implies a greater propensity for aggregation.


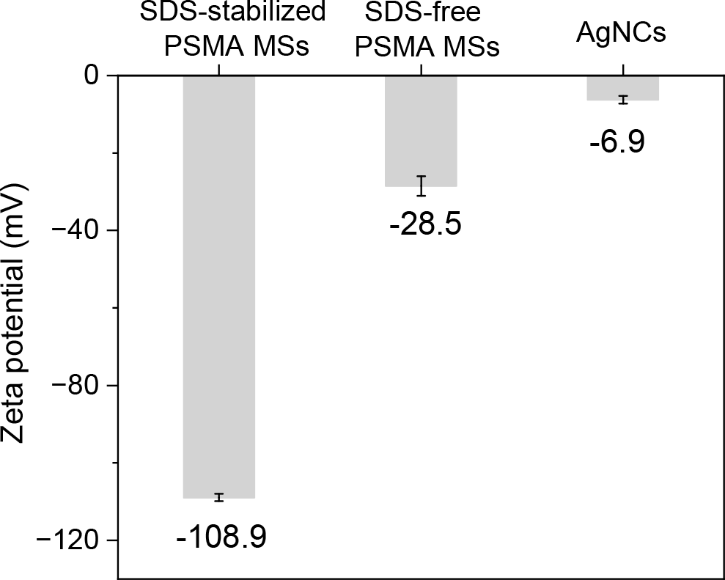


**Figure S22.** Zeta potentials of SDS-stabilized PSMA MSs, SDS-free PSMA MSs without SDS, and AgNCs.


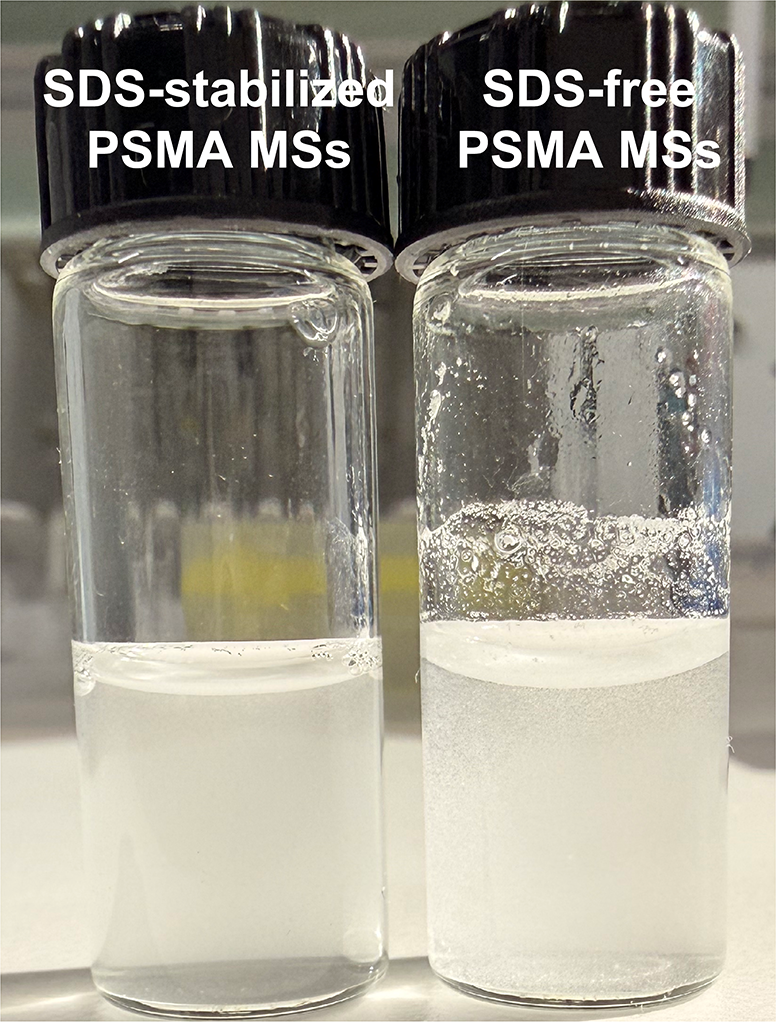


**Figure S23**. Photographs of SDS-stabilized PSMA MSs and SDS-free PSMA MSs in aqueous solution.


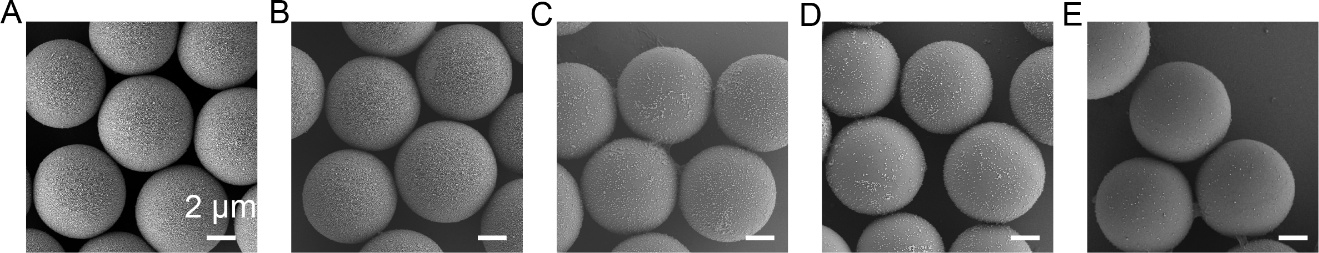


**Figure S24.** Effect of solvent polarity on co-assembly of PSMA MSs and AgNCs. The volume percentage of 2-propanol in water (*V*_IPA_). (A) 0%, (B) 25%, (C) 50%, (D) 75%, (E) 100%. The coverage rate decreased with decreasing solvent polarity.


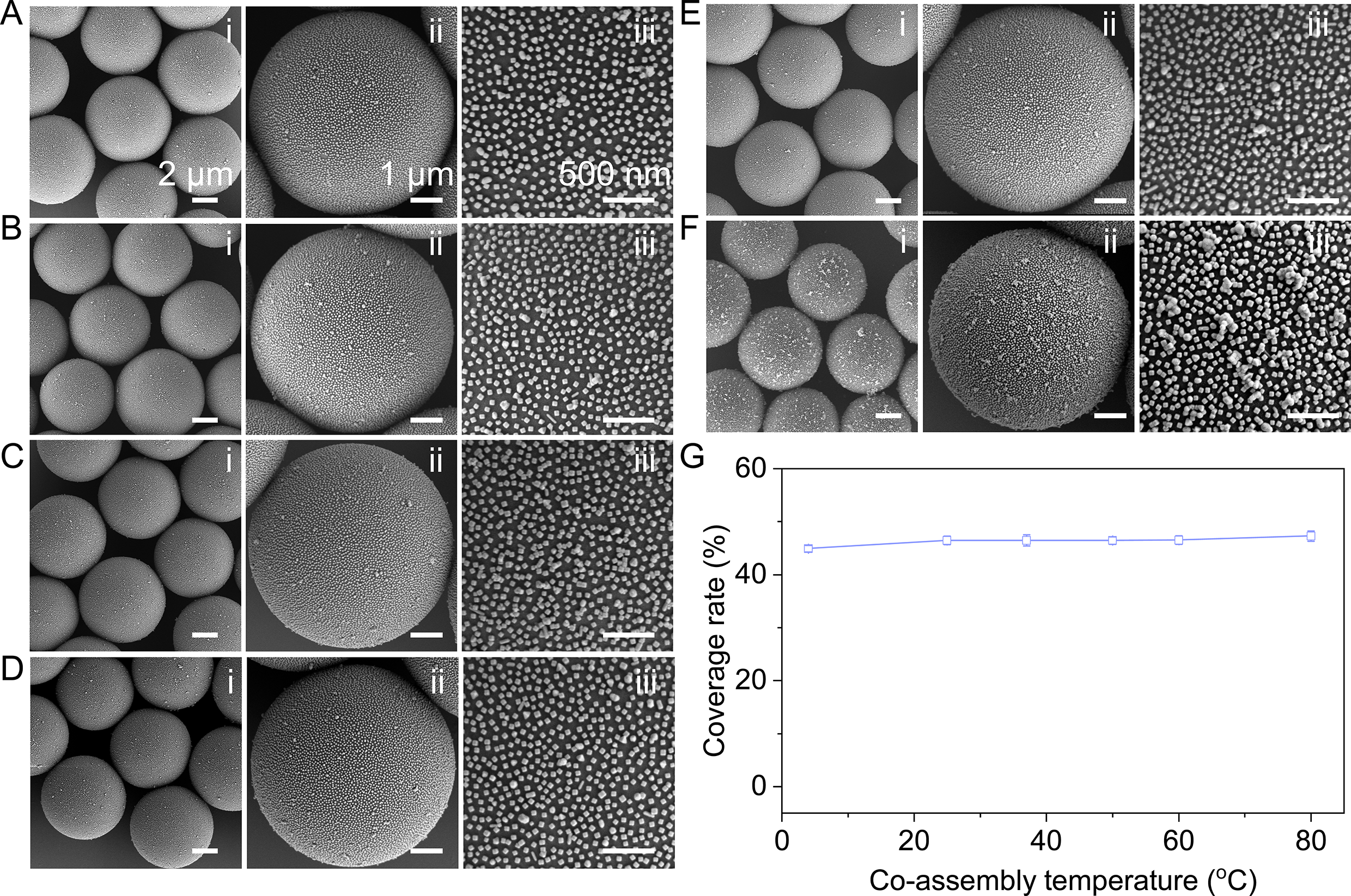


**Figure S25.** Temperature on co-assembly. (A) 4 °C, (B) 25 °C, (C) 37 °C, (D) 50 °C, (E) 60 °C, and (F) 80 °C. (G) The coverage rates at different temperatures. The abundance of AgNCs co-assembled onto PSMA MSs at a wide range from 0-80 °C remained consistent over 44.9%.


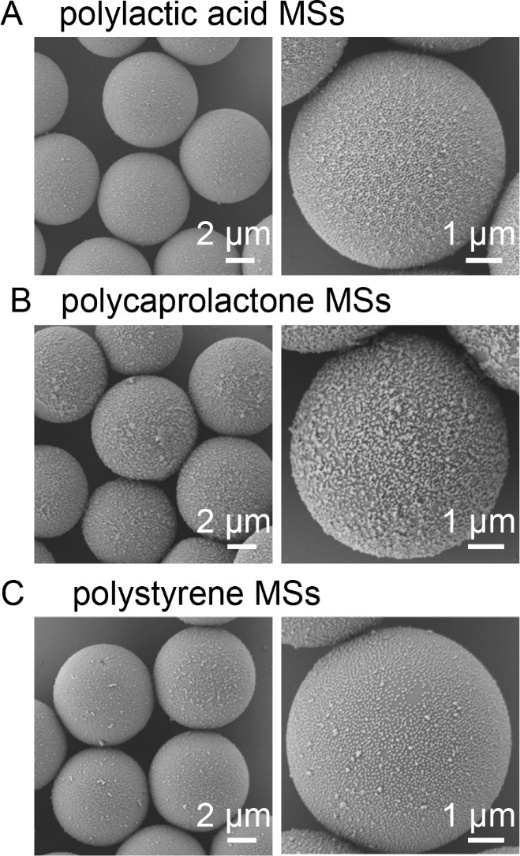


**Figure S26.** SEM analysis of co-assembly between AgNCs and other polymer MSs, including polylactic acid MSs (A), polycaprolactone MSs (B), and polystyrene MSs (C).

**
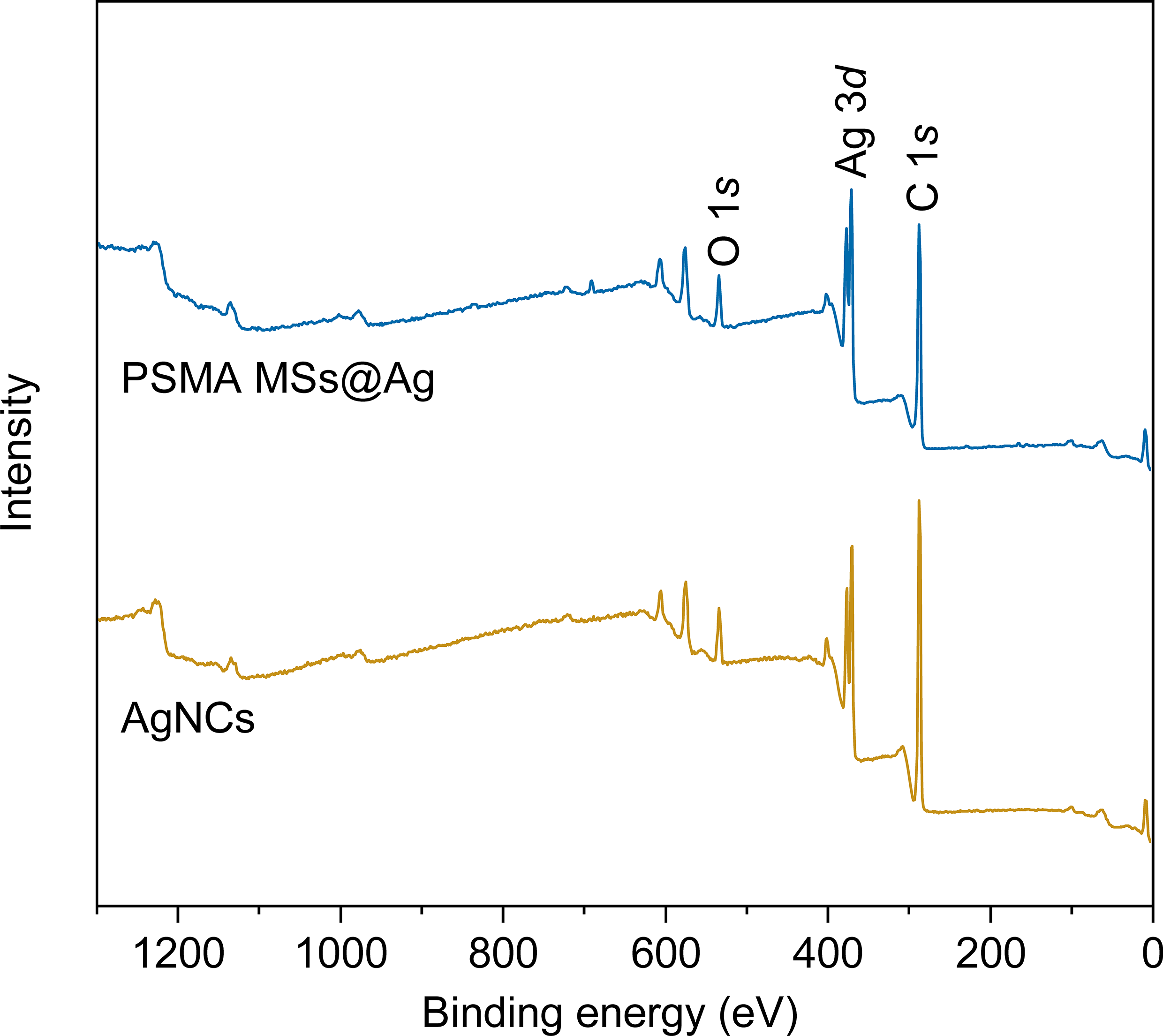
**

**Figure S27.** XPS spectra of the AgNCs and PSMA MSs@Ag.


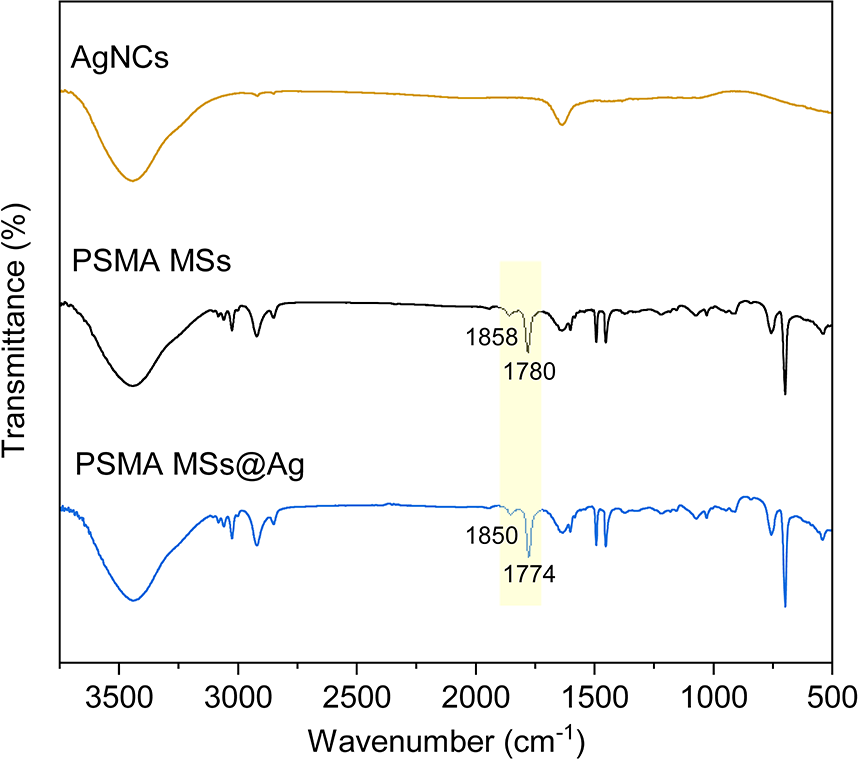


**Figure S28.** FTIR spectra of AgNCs, PSMA MSs, and PSMA MSs@Ag.

**
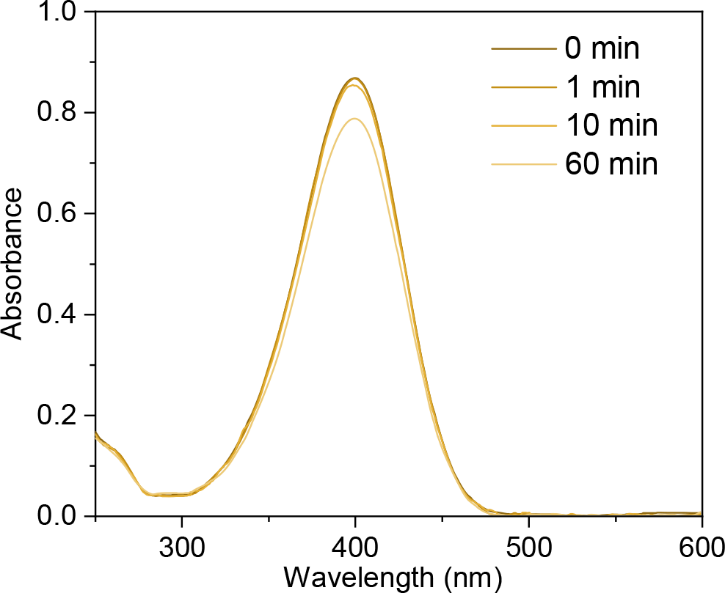
**

**Figure S29.** UV-vis spectra of 4-NP reduction without mag-MSs@Ag. The small intensity variation of the peak implies that the reaction was making very little progress during the observed period.


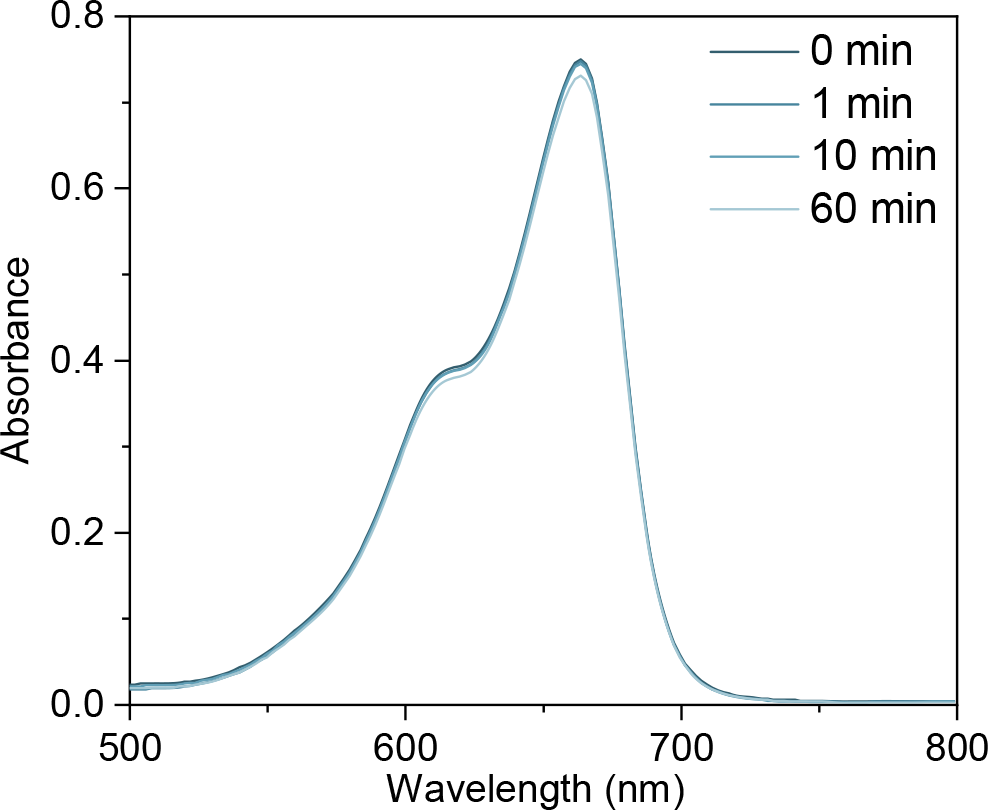


**Figure S30.** UV-vis spectra of MB reduction without mag-MSs@Ag. Minimal changes in the characteristic MB absorbance peak intensity were observed over the monitored period, indicating the reaction progress was slow.


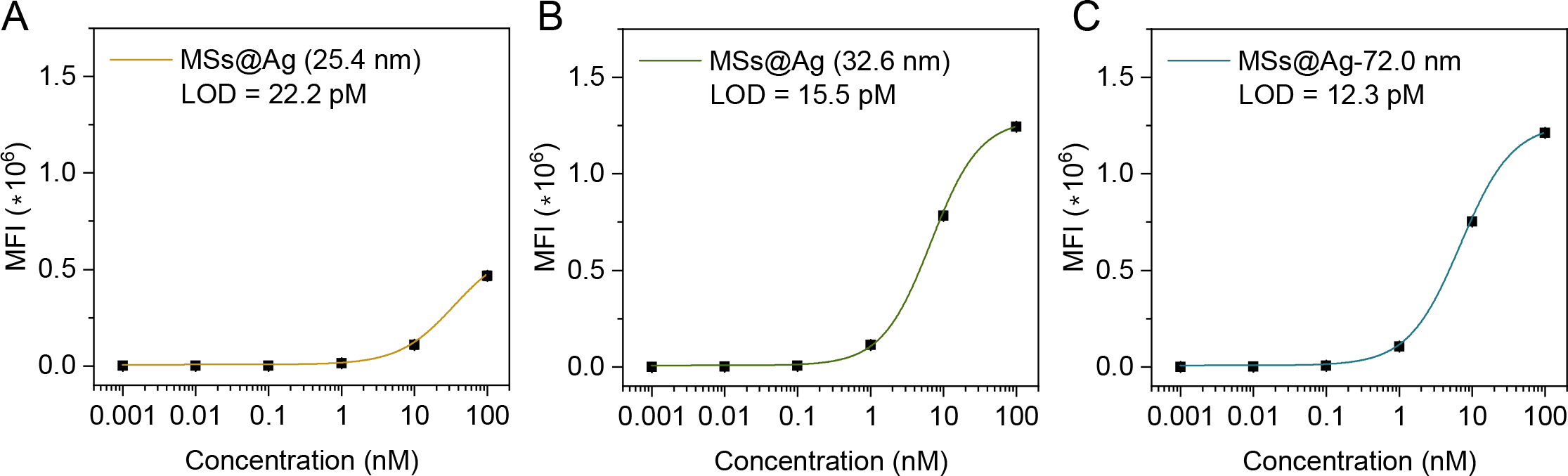


**Figure S31.** Standard curves and detection limits for MSs@Ag. (A) MSs@Ag-25.4 nm, (B) MSs@Ag-32.6 nm, (C) MSs@Ag-72.0 nm. Limits of detection (LOD): 22.2 pM (A), 15.5 pM (B), 12.3 pM (C).


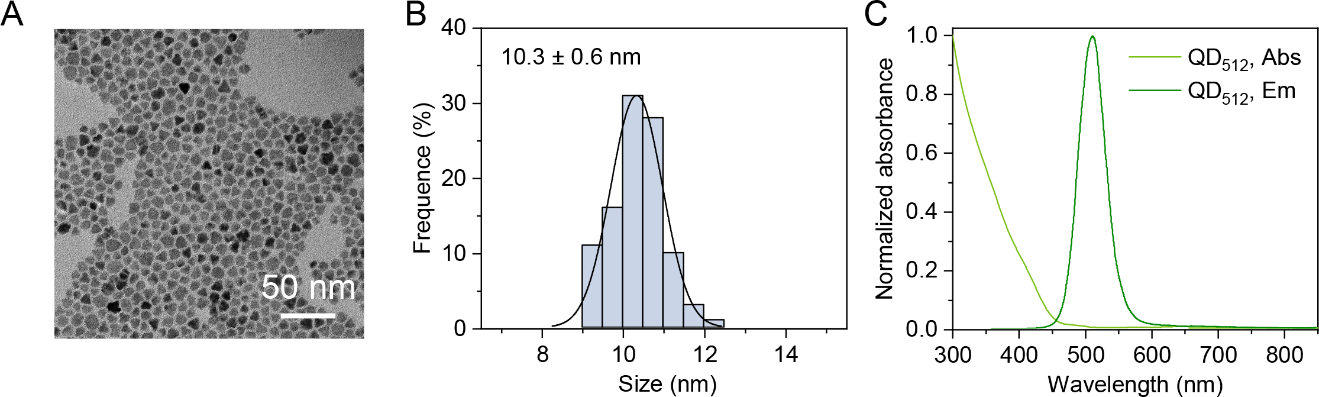


**Figure S32.** TEM image (A), particle-size distribution with an average diameter of ~ 10.3 nm (B), and absorption and emission spectra (C) of QD_512_.


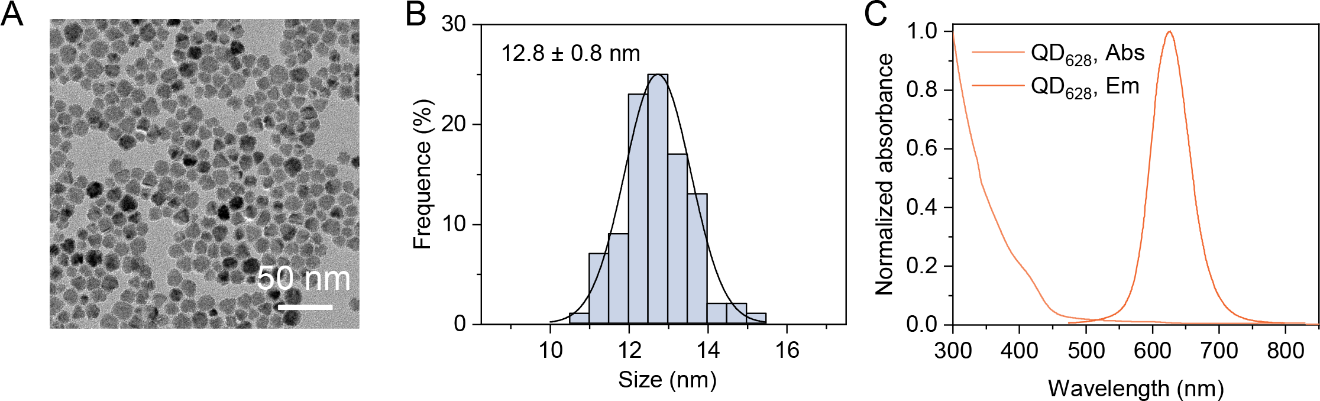


**Figure S33.** TEM image (A), particle-size distribution with an average diameter of ~ 12.8 nm (B), and absorption and emission spectra (C) of QD_628_.


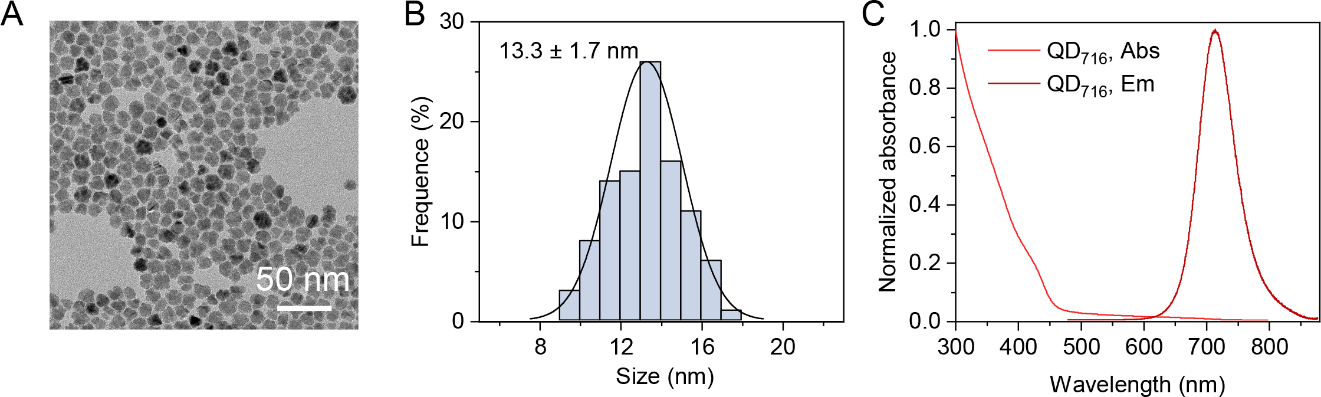


**Figure S34.** TEM image (A), particle-size distribution with an average diameter of ~ 13.3 nm (B), and absorption and emission spectra (C) of QD_716_.


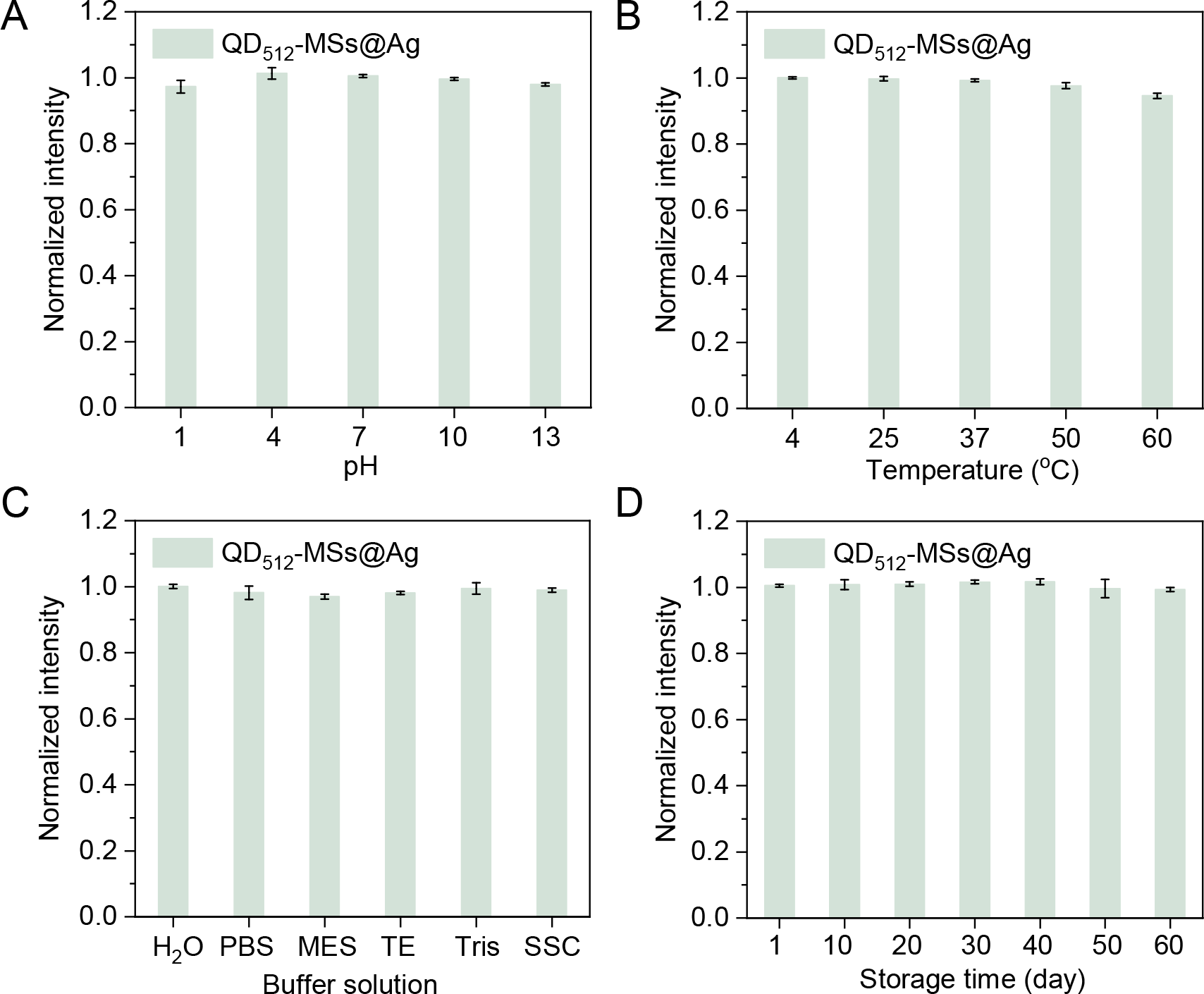


**Figure S35.** Fluorescence stability of QD_512_-MSs@Ag under different environmental conditions. (A) pH stability. (B) Thermal stability. (C) Buffer solution stability. (D) Long-term stability. Minimal fluorescence variation (< 6% intensity change) is observed across all conditions, indicating that QD_512_-MSs@Ag have good fluorescence stability.


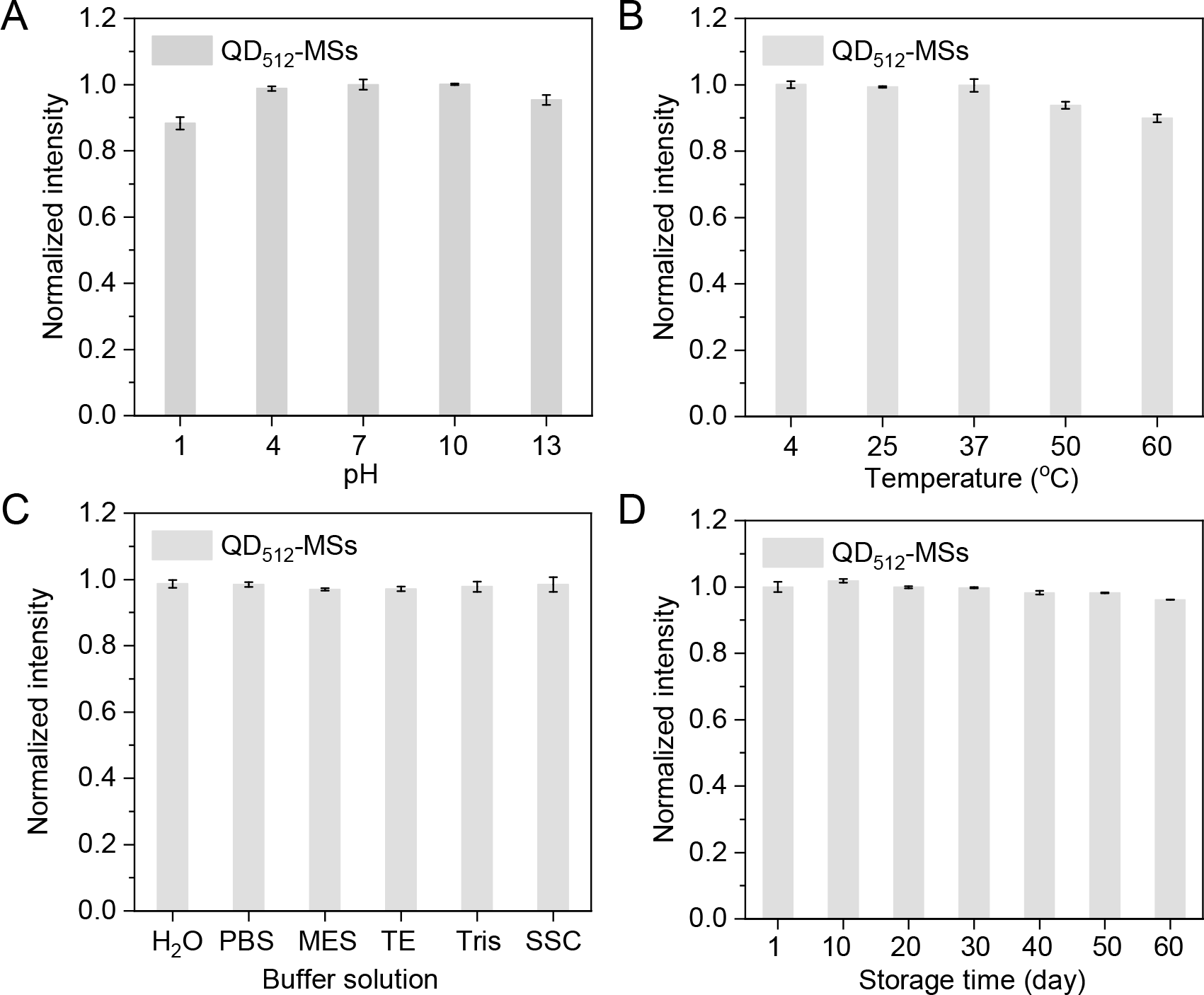


**Figure S36.** Fluorescence stability of QD_512_-MSs under different environmental conditions. (A) pH stability. (B) Thermal stability. (C) Buffer solution stability. (D) Long-term stability. The fluorescence intensity decreased by 11.7% at pH = 1 and by 10.2% at 60 °C.


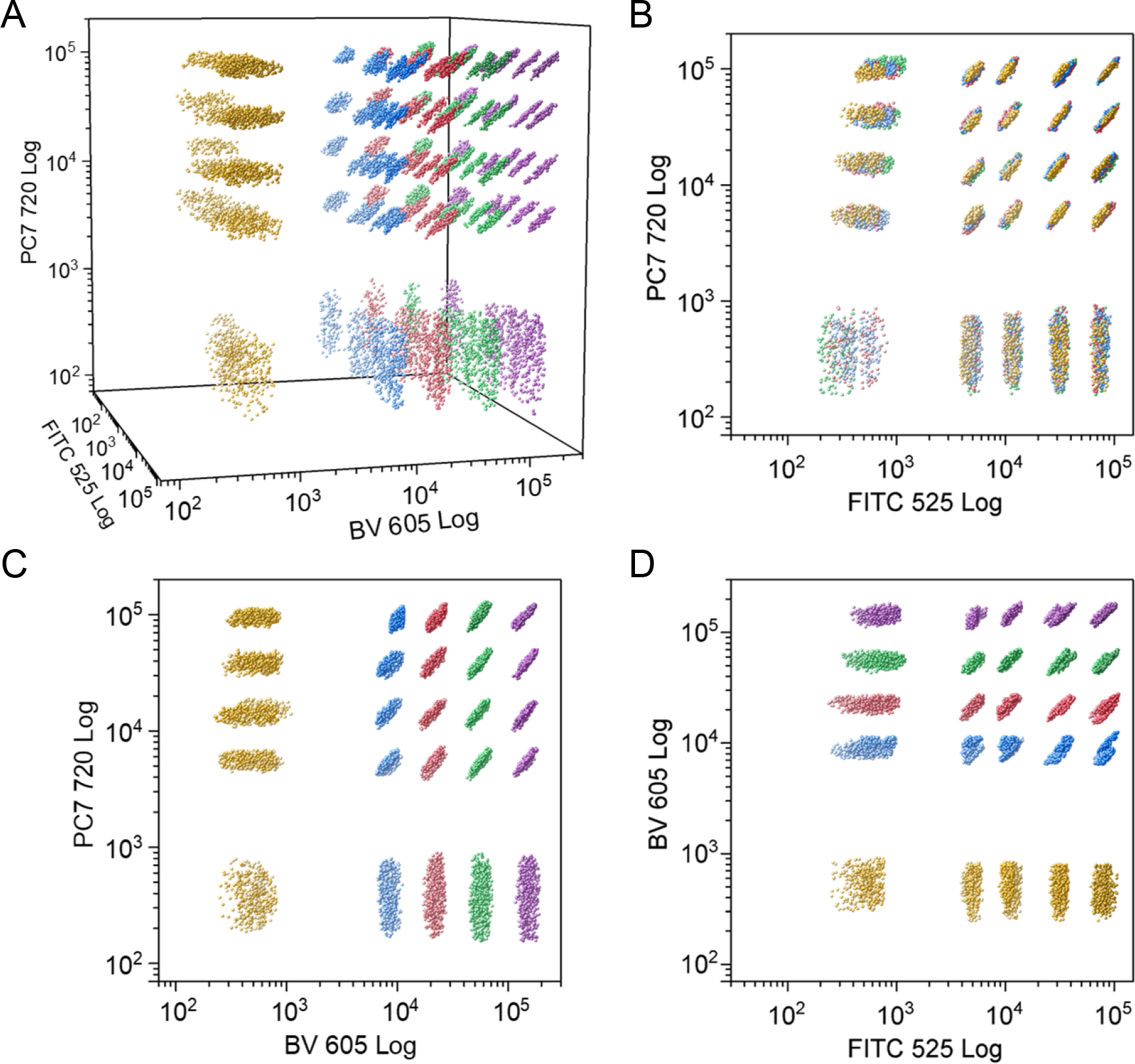


**Figure S37.** Three-dimensional (3D) QDs-based barcode matrix array. (A) Combinatorial library matrix (124 codes) based on three color QDs (QD_512_, QD_628,_ and QD_716_) and intensity (5 levels). (B) Front, (C) side, and (D) top projections of encoded QDs-MSs.


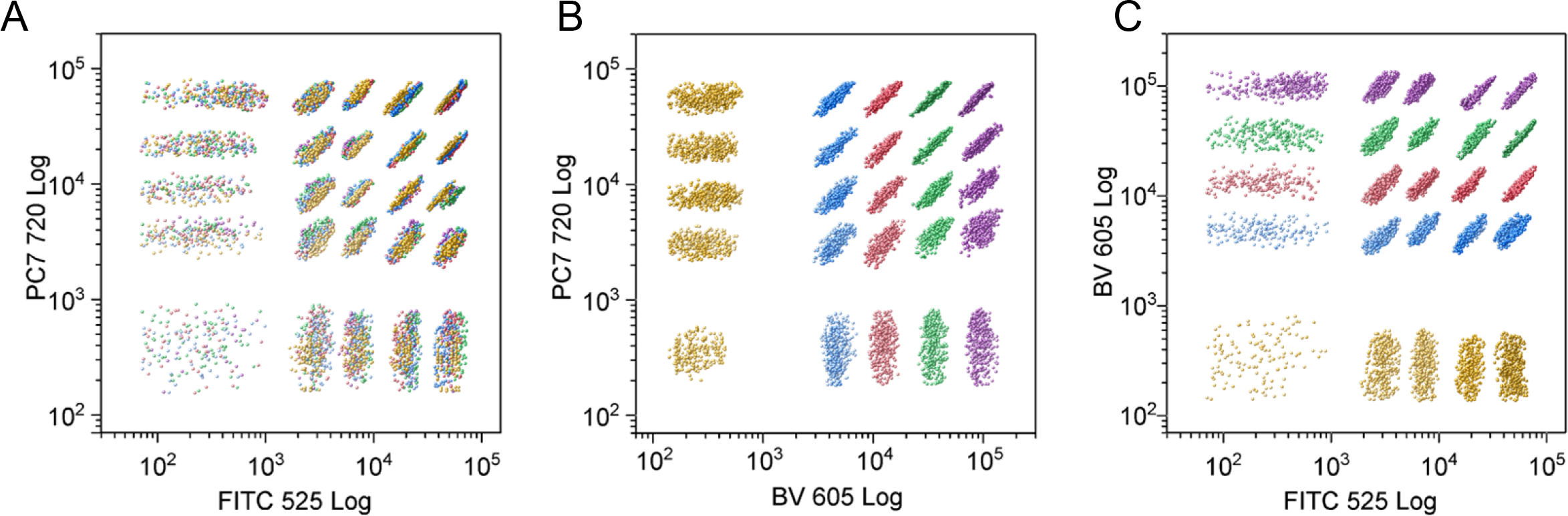


**Figure S38.** (A) Front, (B) side and (C) top projection views of the Ag-decorated QDs-based barcode matrix array.


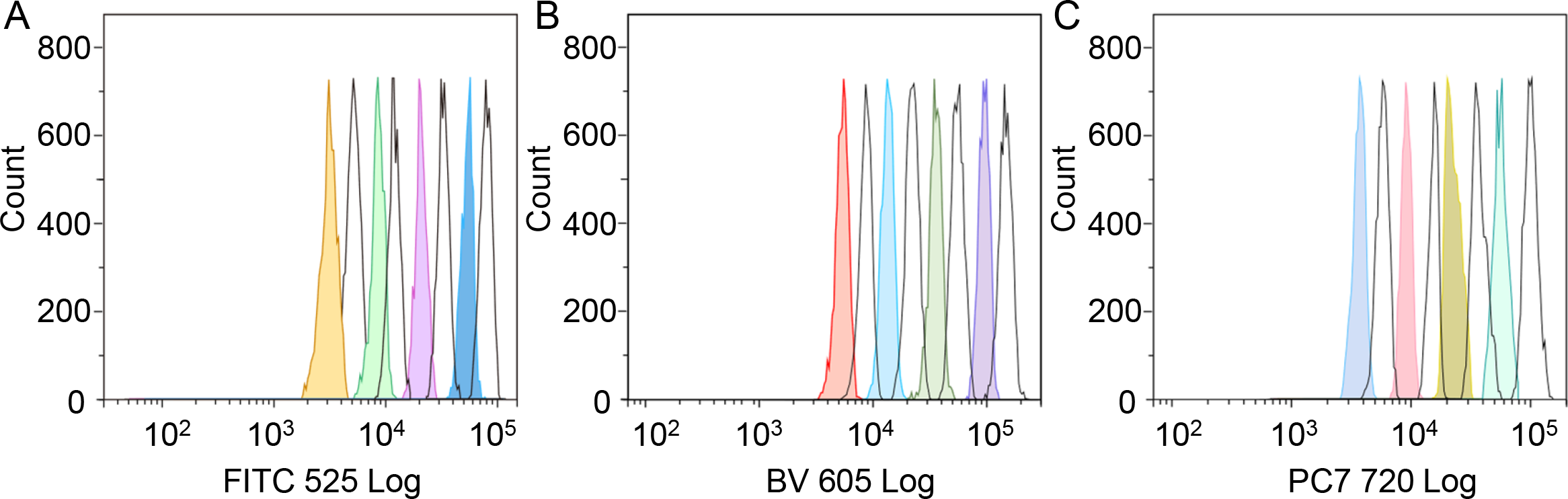


**Figure S39.** Fluorescence intensity histograms of QDs-MSs (colorless) and QDs-MSs@Ag (filled color) for (A) QD_512_, (B) QD_628_, and (C) QD_716_. The Ag-nanoshell coating caused a slight decrease in fluorescence intensity due to photon blocking, but the clusters were still separated from each other.


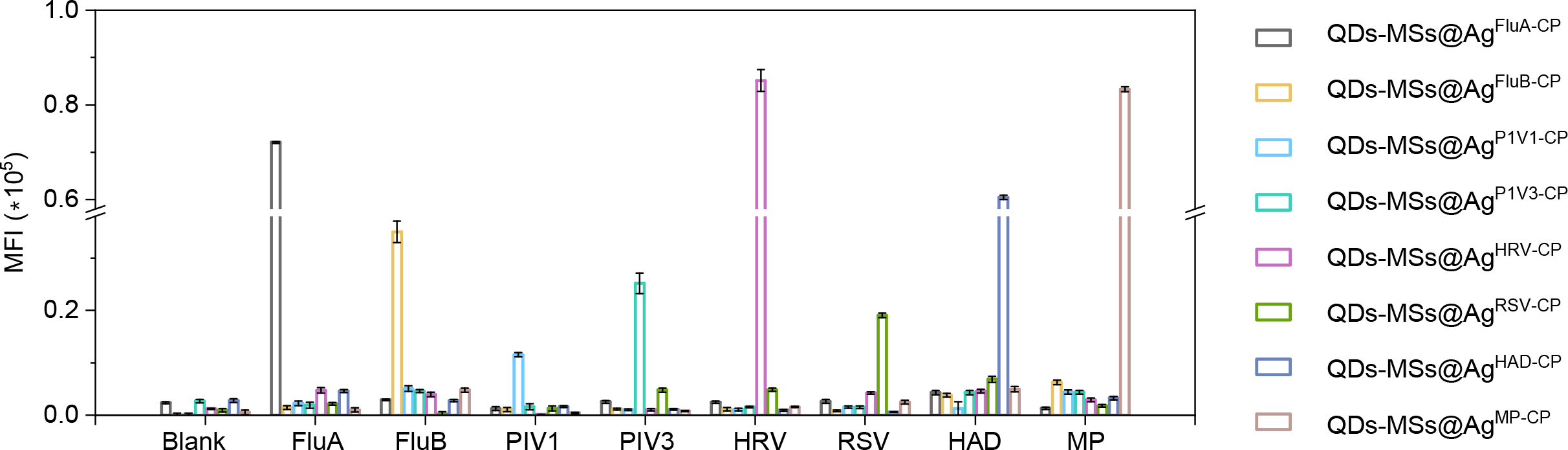


**Figure S40.** Specificity evaluation of eight QDs-MSs@Ag barcodes for the corresponding respiratory viruses. The high-intensity PE signals with the MFI values were detected only when the QDs-MSs@Ag barcodes were selectively targeted with the specific viral RNA sequences.


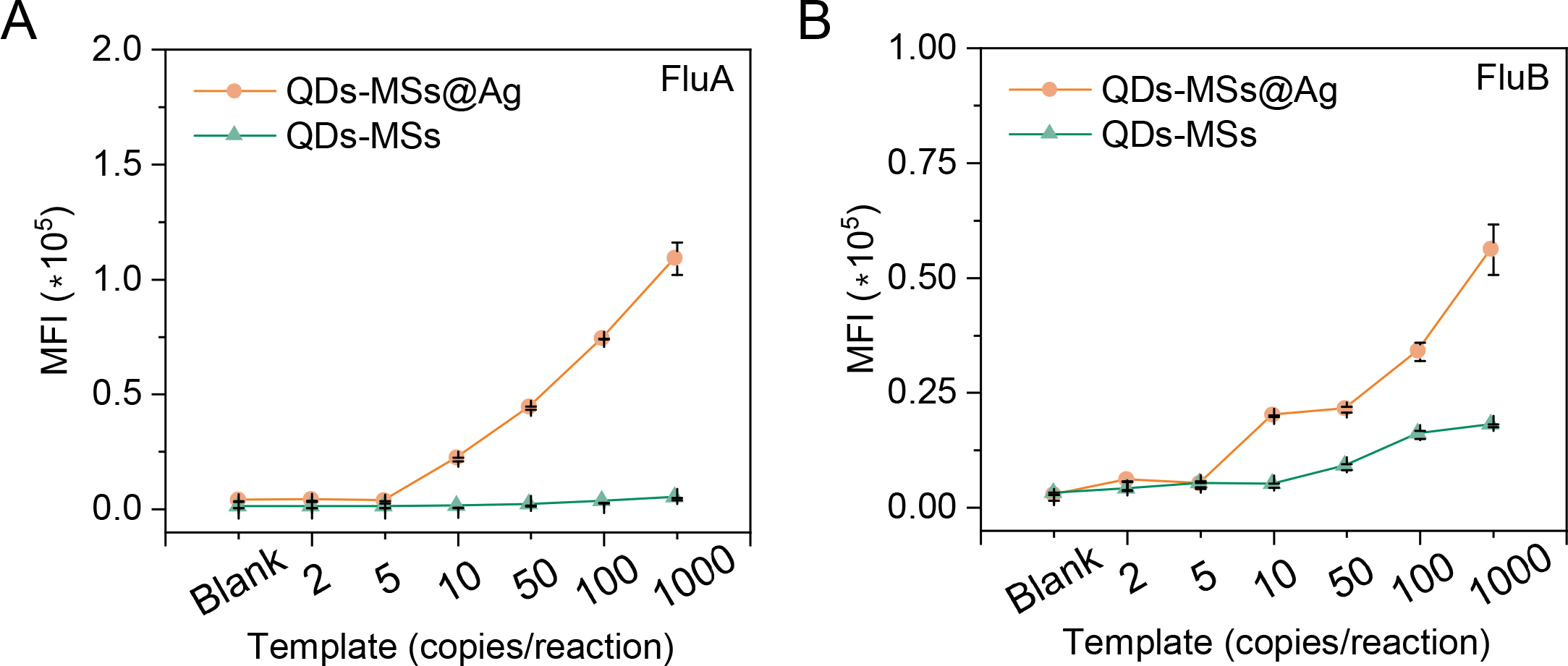


**Figure S41.** Sensitivity comparison for FluA (A) and FluB (B) detection with QDs-MSs and QDs-MSs@Ag. The QDs-MSs@Ag achieved a lower limit of detection (LOD = 10 copies/reaction) compared to QDs-MSs (LOD = 50 copies/reaction), showing improved sensitivity.

**Table S1.** Atomic composition of PSMA MSs without SDS determined by EDS.

| Element | At. No. | Line series | Mass Norm.[%] | Atom [%] |
| --- | --- | --- | --- | --- |
| C | 6 | K | 96.81 | 97.60 |
| O | 8 | K | 3.14 | 2.38 |
| Na | 11 | K | 0.01 | 0.00 |
| S | 16 | K | 0.03 | 0.01 |
|  |  |  | 100.00 | 100.00 |

(Detection limit: 0.1 at%. Absence of Na and S signals confirms complete removal of SDS surfactant.)

**Table S2.** Atomic composition of SDS-stabilized PSMA MSs determined by EDS.

| Element | At. No. | Line series | Mass Norm. [%] | Atom [%] |
| --- | --- | --- | --- | --- |
| C | 6 | K | 93.42 | 96.13 |
| O | 8 | K | 2.90 | 2.24 |
| Na | 11 | K | 1.37 | 0.74 |
| S | 16 | K | 2.31 | 0.89 |
|  |  |  | 100.00 | 100.00 |

**Table S3.** Comparison of the catalytic activity of different catalysts for 4-NP/MB reduction.

| **Catalyst** | **Pollutant** | **n (mmol)** | **Amount of catalyst (mg)** | **Time (min)** | **Cycle number** | **References** |
| --- | --- | --- | --- | --- | --- | --- |
| Fe_3_O_4_@PPy-MAA/Ag | 4-NP | 1.0 | 7.5 | 25 | 8 | Applied Catalysis B: Environmental 2019, 244, 546-558. |
| Urchin-like Fe_3_O_4_@PDA-Ag | MB | 1.25 × 10^-6^ | 5 | 4 | 5 | J. Hazard. Mater. 2018, 350, 66-75. |
| Au NP-decorated micellar brushes | 4-NP | 1.2 × 10^-4^ | / | 2 | / | Angew. Chem. Int. Ed. 2021, 60, 24637-24643. |
| Fe_3_O_4_@SiO_2_-Au@mSiO_2_ | 4-NP | 5 × 10^-4^ | 3.0 | 12 | 8 | J. Am. Chem. Soc. 2010, 132, 8466-8473. |
| O/W SiO_2_ NP-AgNP Pickering emulsions | 4-NP | 8 × 10^-4^ | 0.063%, wt | 15 | / | Nat. Commun. 2023, 14, 1392. |
| TiO_2_/Pd microspheres | 4-NP | 3 × 10^-4^ | 0.7 | 20 | 4 | Angew. Chem. Int. Ed. 2012, 51, 6406-6410. |
| Fe_3_O_4_@AMALG12@Ag | 4-NP | 2.5× 10^-3^ | 30 | 3.5 | / | Chem. Eng. J. 2023, 453, 139593. |
| **mag-MSs@Ag** | **4-NP** | **3 × 10^-4^** | **1.5** | **3** | **15** | **Our work** |
| **mag-MSs@Ag** | **MB** | **4.5 × 10^-5^** | **1.5** | **3** | **15** | **Our work** |

**Table S4.** Sequences of DNA strands with different lengths.

| Name | Sequence (5’ to 3’) |
| --- | --- |
| 18 bp capture ssDNA | SH-TTTTAGAGATATGAGCAG |
| 18 bp targeted ssDNA | biotin-CTGCTCATATCTCTAAAA |
| 45 bp capture ssDNA | SH-TTTTAGAGATATGAGCAGAACTGGAAAGGAGGCTGAGAGATG  GCT |
| 45 bp targeted ssDNA | biotin-AGCCATCTCTCAGCCTCCTTTCCAGTTCTGCTCATATCTCTA  AAA |
| 84 bp capture ssDNA | SH-TTTTAGAGATATGAGCAGAACTGGAAAGGAGGCTGAGAGAT  GGCTCGAGTACTACCAGGCTGCGACTCGTCAGACGTATAGTGA |
| 84 bp targeted ssDNA | biotin-TCACTATACGTCTGACGAGTCGCAGCCTGGTAGTACTCGA  GCCATCTCTCAGCCTCCTTTCCAGTTCTGCTCATATCTCTAAAA |

**Table S5.** Comparison of the coding capacity of plasmonic-fluorescent encoded array.

| **Plasmonic-fluorescent encoded array** | **Fluorescence** | **Metal** | **Encoding capacity** | **References** |
| --- | --- | --- | --- | --- |
| Plasmonic gold beads | Cy3 and Alexa 488 | AuNPs | 6 | Chem. Sci. 2014, 5, 4070-4075. |
| Silver nanoshell-coated QD microbeads | QD_510_, QD_575_ and QD_665_ | AgNPs | 3 | Nano Today 2013, 8, 228-234. |
| CYs@nGMs | SRh101 and Rh110 | AuNPs | 30 | Chem. Eng. J. 2025, 513, 162850. |
| **QDs-MSs@Ag** | **In P QD_512_, QD_628_, and QD_716_** | **AgNCs** | **124** | **Our work** |

**Table S6.** Sequences of capture probes.

| Name | Sequence (5’ to 3’) |
| --- | --- |
| FluA-CP | SH-GCCGAGATCGCGCAGAGACT |
| FluB-CP | SH-AGCCAATTCGAGCAGCTG |
| PIV1-CP | SH-GTAATAGCTGCAGGAACAAG |
| PIV3-CP | SH-AATAATATTTCTCGGGTATGGAG |
| HRV-CP | SH-GTCGTAATGAGCAATTGCGG |
| RSV-CP | SH-TTCACGAAGGCTCCACAT |
| HAD-CP | SH-TTCCTGCTCTCACAGATCA |
| MP-CP | SH-GACAACAGCGCTAAGG |

**Table S7.** Primer sequences of 8 respiratory viruses.

| Target | Primers | Sequence (5’ to 3’) |
| --- | --- | --- |
| FluA | FluA-F | CTAACCGAGGTCGAAACGT |
|  | FluA-R | biotin-GGTCTTGTCTTYARCCACTCCAT |
| FluB | FluB-F | TCCTCAACTCACTCTTCGAG |
|  | FuB-R | biotin-CGGTGCTCTTGACCAAATTGG |
| PIV1 | PIV1-F | CAACGACAACAGGAAATCATGTTCTG |
|  | PIV1-R | biotin-GGATGGTCAAAGGTTATATCTTCATT |
| PIV3 | PIV3-F | CTGTTGGACCAGGGATATACTAC |
|  | PIV3-R | biotin-CGGGACACCCAGTTGTGTTGCAG |
| HRV | HRV-F | TCCTCCGGCCCCTGAATG |
|  | HRV-R | biotin-AAACACGGACACCCAAAGTAGT |
| RSV | RSV-F | ATCTATCTGCCAATCAGCCAACCAG |
|  | RSV-R | GGCACCCATATTGTWAGTGATGC |
| HAD | HAD-F | ACCATCACCACCGTCAGTG |
|  | HAD-R | biotin-CGGCGTCTGGCGTCAGTA |
| MP | MP-F | CCGGGGCGTGGGCCTTAGT |
|  | MP-R | biotin-CGAGGTCATACCGGCGTAAC |

**Movie S1 (separate file).** Local electric field of AgNCs on the PSMA MSs. Finite-difference time-domain (FDTD) simulations revealed that the immobilization of AgNCs on PSMA MSs results in a significantly intensified electric field.
